# An Adaptive Threshold in Mammalian Neocortical Evolution

**DOI:** 10.1371/journal.pbio.1002000

**Published:** 2014-11-18

**Authors:** Eric Lewitus, Iva Kelava, Alex T. Kalinka, Pavel Tomancak, Wieland B. Huttner

**Affiliations:** 1 Max Planck Institute of Molecular Cell Biology and Genetics, Dresden, Germany; CAS-MPG Partner Institute for Computational Biology, China

## Abstract

Expansion of the neocortex is a hallmark of human evolution. However, determining which adaptive mechanisms facilitated its expansion remains an open question. Here we show, using the gyrencephaly index (GI) and other physiological and life-history data for 102 mammalian species, that gyrencephaly is an ancestral mammalian trait. We find that variation in GI does not evolve linearly across species, but that mammals constitute two principal groups above and below a GI threshold value of 1.5, approximately equal to 10^9^ neurons, which may be characterized by distinct constellations of physiological and life-history traits. By integrating data on neurogenic period, neuroepithelial founder pool size, cell-cycle length, progenitor-type abundances, and cortical neuron number into discrete mathematical models, we identify symmetric proliferative divisions of basal progenitors in the subventricular zone of the developing neocortex as evolutionarily necessary for generating a 14-fold increase in daily prenatal neuron production, traversal of the GI threshold, and thus establishment of two principal groups. We conclude that, despite considerable neuroanatomical differences, changes in the length of the neurogenic period alone, rather than any novel neurogenic progenitor lineage, are sufficient to explain differences in neuron number and neocortical size between species within the same principal group.

## Abstract 2

A study of the evolutionary history of cortical folding in mammals, its relationship to physiological and life-history traits and the underlying cortical progenitor behavior during embryogenesis, explains the diversity of folding we see across modern mammals. The diversity of neocortical folding among mammals can be explained by two distinct neurogenic programs, which give rise to mammals with a highly folded neocortex and mammals with slightly folded or unfolded neocortex, each occupying a distinct ecological niche.

## Author Summary

What are the key differences in the development and evolution of the cerebral cortex that underlie the differences in its size and degree of folding across mammals? Here, we present phylogenetic evidence that the Jurassic era mammalian ancestor may have been a relatively large-brained species with a folded neocortex. We then show that variation in the degree of cortical folding (gyrencephaly index [GI]) does not evolve linearly across species, as previously assumed, but that mammals fall into two principal groups associated with distinct ecological niches: low-GI mammals (such as mice and tarsiers) and high-GI mammals (such as dolphins and humans), which are found to generate on average 14-fold more brain weight per day of gestation. This greater daily brain weight production in mammals with a highly folded neocortex requires a specific class of progenitor cell-type to adopt a special mode of cell division, which is absent in mammals with slightly folded or unfolded neocortices. Differences among mammals within the same GI group (high or low) are not due to different programming, but rather the result of differences in the length of the neurogenic period. So, the impressively large and folded human neocortex, which is three times the size of the chimpanzee neocortex, can be explained by a modest evolutionary extension of the neurogenic period with respect to its closest primate ancestors.

## Introduction

Development of the mammalian, and in particular human, neocortex involves various types of neural stem and progenitor cells that reside in the germinal layers of the cortical wall [1]–[5]. An increase in the proliferative capacity of these cells underlies the evolutionary expansion of the neocortex, notably the increase in neuron number. At the onset of mammalian cortical neurogenesis, neuroepithelial cells transform into apical radial glia (aRG), which repeatedly undergo mitosis at the apical surface of the ventricular zone (VZ) and typically divide asymmetrically to self-renew and generate either a neuron, an apical intermediate progenitor cell, a basal intermediate progenitor cell (bIP), or basal radial glia (bRG) (the latter two being collectively referred to as basal progenitors [BPs]) [1]–[5].

In contrast to aRG cells, BPs delaminate from the apical surface and translocate their nucleus to the basal-most region of the VZ to form a secondary germinal layer, the subventricular zone (SVZ), where they divide symmetrically or asymmetrically. In developing mouse neocortex, bIPs typically divide symmetrically to generate two post-mitotic neurons (neurogenic bIP) [5]–[8], whereas in the macaque and human, bIPs can also frequently undergo symmetric proliferative divisions (proliferative bIP) [5],[9],[10]. Similarly to aRG cells in the VZ, bRG cells in the SVZ divide both symmetrically and asymmetrically [9],[11]–[14], which leads to the proliferation of their population and their self-renewal, respectively [5]. Importantly, the symmetric proliferative divisions of bIPs and bRG cells result in the transit-amplification of BPs [9],[10],[12],[15],[16], which in turn allows for an increase in the efficiency of subsequent neuron generation [2],[5],[17],[18].

In mammals exhibiting an abundance of BPs during cortical neurogenesis, the SVZ becomes further compartmentalized into an inner (ISVZ) and outer SVZ (OSVZ), as first described in the macaque [19] and subsequently observed in several species in which bRG cells constitute a relatively high proportion of BPs [1]–[3],[5]. Moreover, bRG cells are characterized by radial fibers, which distinguish them from bIPs. These radial fibers of bRG cells in the OSVZ of gyrencephalic mammals typically have divergent, rather than parallel, trajectories to the cortical plate, which is thought to contribute to creating the folded cortical pattern observed in these species through the tangential expansion of migrating neurons [2],[3],[5],[20]. For this reason, and based on supporting evidence obtained in the gyrencephalic human and ferret and lissencephalic mouse, an abundance of asymmetrically dividing bRG cells in the OSVZ has been thought to be necessary for establishing a relatively large and gyrencephalic neocortex [1],[9],[11],[12]. However, subsequent work in the lissencephalic marmoset (*Callithrix jacchus*) has shown that bRG cells may, in fact, exist in comparable abundance in the developing neocortex of both gyrencephalic and lissencephalic species [21],[22], indicating that bRG abundance alone cannot be sufficient for either establishing or increasing cortical gyrification. Rather, the mode of cell division, that is, symmetric proliferative versus asymmetric self-renewing, of bRG cells, and of BPs in general, may be a critical determinant of the extent to which gyrification occurs and the neocortex expands. Notably, despite considerable progress in the study of brain size evolution [23]–[25], the adaptive mechanism that has evolved along certain mammalian lineages to produce a large and folded neocortex is not known.

In this study, we analyzed physiological and life-history data from 102 mammalian species (Tables S1 and S2; Database S1). We show that a gyrencephalic neocortex is ancestral to all mammals and that GI, like brain size, has increased and decreased along many mammalian lineages. These changes may be reliably characterized by convergent adaptations into two distinct physiological and life-history programs, resulting in a bimodal distribution of mammalian species with regard to the gyrencephaly index (GI) and the amount of brain weight produced per gestation day. We explain the appearance of these two groups in mammalian evolution by the adaptation of differences in the lineages and modes of cell division of progenitor cells during corticogenesis. We predict that symmetric proliferative BP divisions are key to evolutionary changes in gyrification and expansion of the neocortex.

## Results

### The Evolutionary History of Mammalian Gyrencephaly

**Figure 1:**
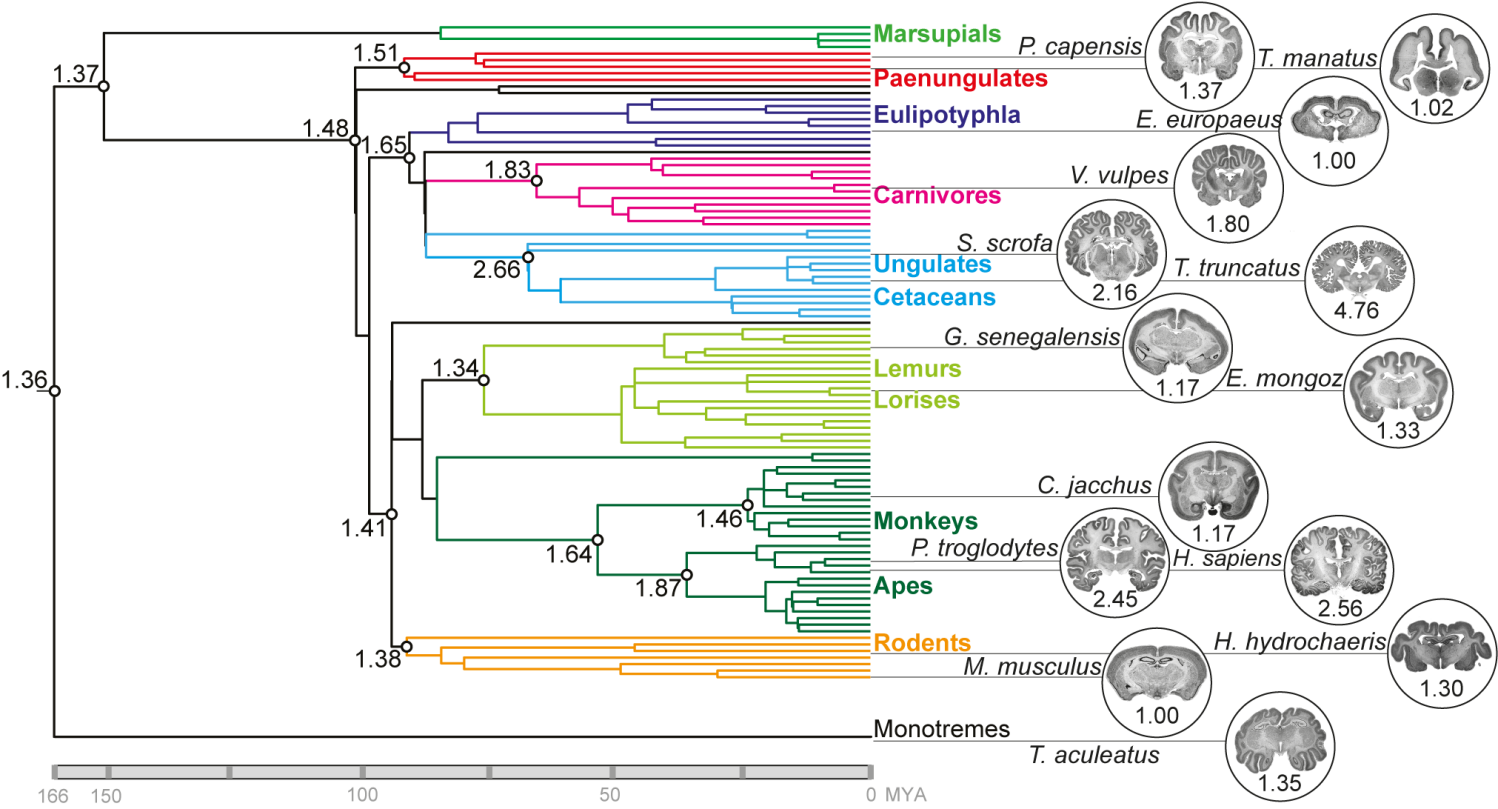
Ancestral reconstruction of GI values for 102 mammalian species. GI values were determined as illustrated in Figure S1 for the species listed in Table S1. Reconstructed GI values for putative ancestors are presented at selected internal nodes of the phylogenetic tree. MYA, million years ago; colors indicate taxonomic groups. Images of Nissl-stained coronal sections of representative species for each taxonomic group (except marsupials) downloaded from http://brainmuseum.org, along with respective GI values, are shown on the right.

We collected GI data (Figure S1) for 102 species sampled from every mammalian order and tested multiple models for GI evolution using a species-level supertree [26]. The model that conferred the most power to explain GI values across the phylogeny while making the fewest assumptions about the data (i.e., that had the lowest Akaike Information Criterion [AIC]) diverged significantly from a null model of stochastic evolution [27] and showed a disproportionate amount of evolutionary change to have occurred recently, rather than ancestrally, in mammals (Figure S2). We identified a folded neocortex (GI = 1.36±0.16 standard error of the mean [SEM]) as an ancestral mammalian trait (Figure 1). This result held even when additional hypothetical lissencephalic species were added to the root of the phylogenetic tree (Table S3).

It is apparent from ancestral and other internal node reconstructions (Figure S3) not only that GI is very variable, but also that reductions in the rate at which GI evolves have favored branches leading to decreases in GI (e.g., strepsirrhines and eulipotyphla) and accelerations in that rate have favored branches leading to increases in GI (e.g., carnivores and caviomorphs). A simulation of the average number of total evolutionary transitions between GI values evidences more affinity for transitioning from high-to-low than low-to-high GI values: the majority of high-to-low transitions (58.3%) occurred in species with a GI<1.47; and the fewest transitions (16.7%) occurred across a threshold GI value (see below) of ∼1.5 (Figure S4). This finding indicates that, although there is an evident trend in mammalian history to become increasingly gyrencephalic, the most variability in GI evolution has been concentrated among species below a certain threshold value (GI = 1.5). We therefore present a picture of early mammalian history, contrary to most previous work, but which is gathering evidence through novel approaches [28],[29], that the Jurassic-era mammalian ancestor may, indeed, have been a relatively large-brained (>10 g) species with a folded neocortex.

### GI Is Bimodally Distributed and Supports Two Principal Mammalian Phenotypes

**Figure 2:**
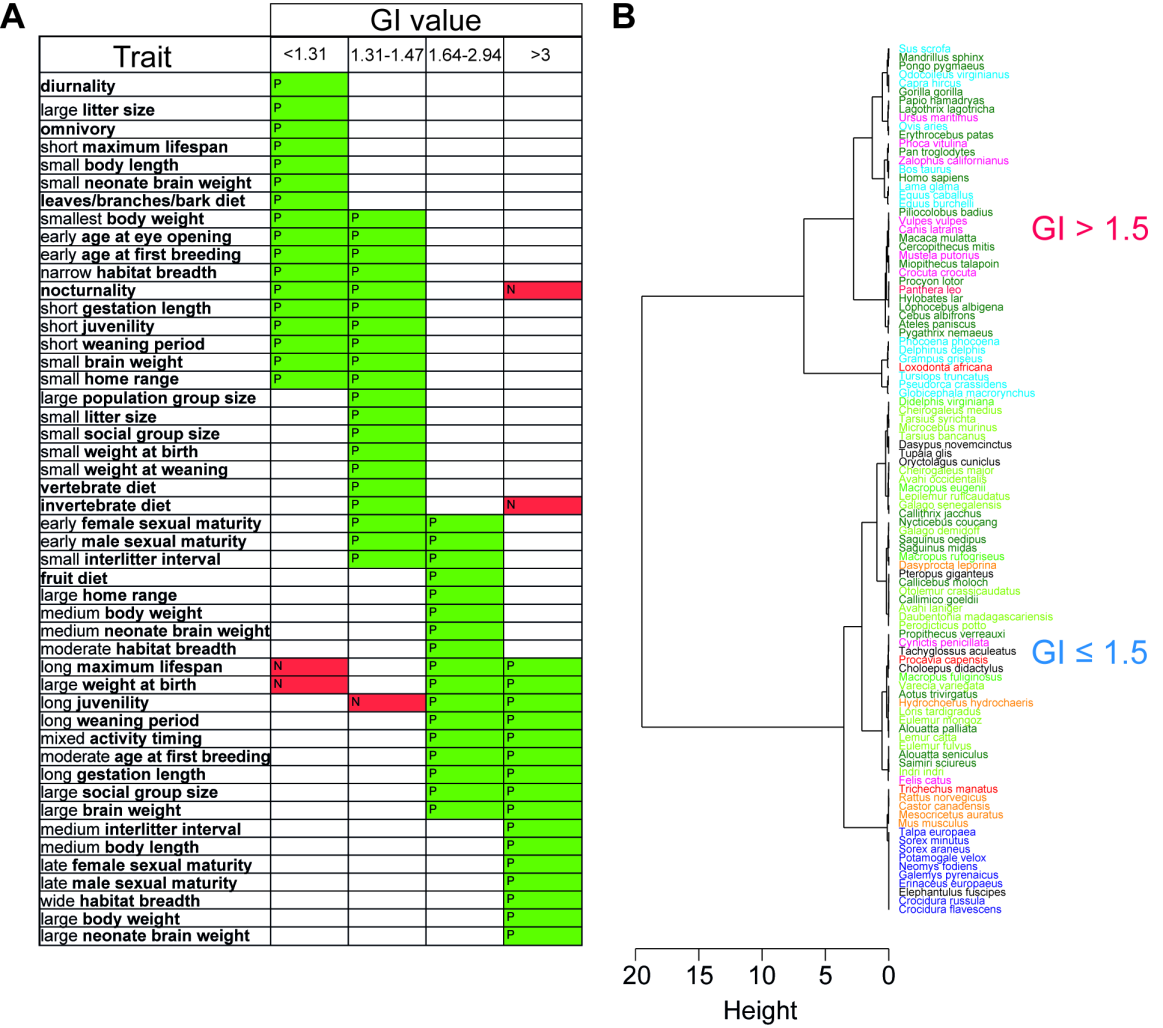
Clustering of GI values based on life-history association analysis (A) and minimum-energy distance (B). (A) Stochastic mapping of physiological and life-history traits with GI values for the 102 mammalian species listed in Table S1. GI values were separated into four groups based on clustering. Thirty-seven traits (bold letters), each comprising two to eight character states (regular letters), were analyzed (see Table S2 for a complete list), and the states showing a significant positive (P, green) or negative (N, red) association with a group of GI values are shown. Traits are listed according to their positive associations with each GI group moving from least to most gyrencephalic. Note the major overlap between the two low-GI groups (10/27) and between the two high-GI groups (9/24), whereas only 3/48 character states are shared between GI groups <1.5 and >1.5. (B) Hierarchical clustering based on minimum-energy distance of the GI values for 101 mammalian species (see Table S1, with *Cynocephalus volans* being omitted from this analysis). Note that the greatest clustering height is between species with GI values ≤1.5 and >1.5. Species of the various taxonomic groups are colored according to Figure 1.

The evolutionary effects of a folded neocortex on the behavior and biology of a species is not immediately clear. We therefore analyzed associations, across the phylogeny, of GI with discrete character states of 37 physiological and life-history traits (Table S2). Distinct sets of small but significant (R^2^≤0.23, *p*<0.03) associations were found for species above and below a GI value of 1.5, indicating that these two groups of species adapt to their environments differently (Figure 2A). Although species above and below GI = 1.5 tend to fall within classical definitions of slow and fast life-histories, respectively, our results argue in favor of a dichotomy rather than a continuum and, additionally, bear out ecological and behavioral associations not historically bracketed in slow or fast life-history paradigms [30]. For example, the result that narrow habitat breadth and large population group size are associated with low-GI (<1.5) species, whereas wide habitat breadth and large social group size are associated with high-GI (>1.5) species, suggests not only that an ecological distinction be made for mammals between the population size of co-habitating individuals and the number of those individuals interacting socially, but also that the number of habitat types in which a species must compete may assert a positive selection pressure on neocortical evolution. Importantly, both the low-GI and high-GI groups are sampled from across the phylogeny, testifying to the absence of a phylogenetic signal in the establishment of the two groups and a functional role for GI in the evolution of life-history programs. Hierarchical clustering analysis also supports a bimodal distribution above and below a GI value of 1.5 (Figures 2B and S5).

### A Neocortical Threshold GI

**Figure 3:**
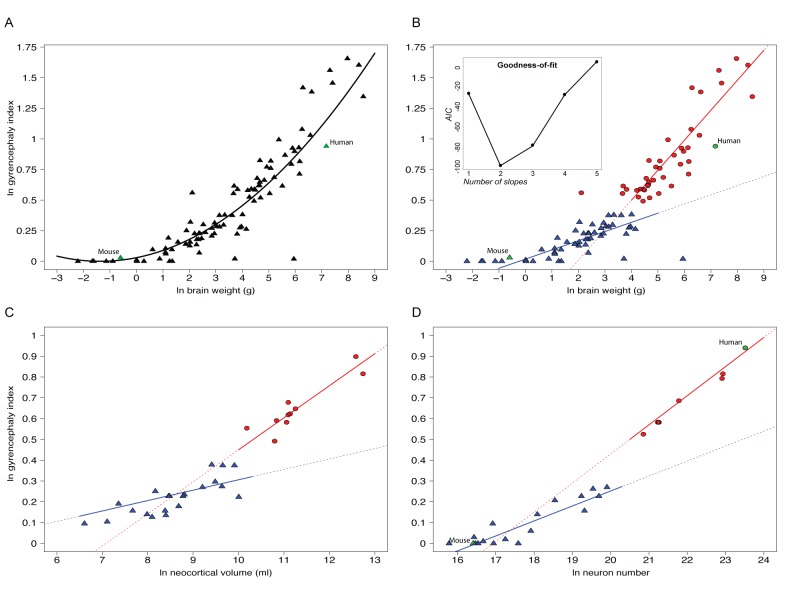
Ln-transformed plots showing GI values as a function of adult brain weight (A, B), neocortical volume (C), and cortical neuron number (D). (A) Regression analysis using one non-linear fit for all values (101 species [see Table S1, with *C. volans* being omitted from this analysis], *y* = 0.018*x* ^2^+0.037*x*+0.014, R^2^ = 0.612, *p* = 6×10^−5^); (B–D) regression analyses using two different linear functions (B, 101 species, blue line: *y* = 0.075*x*−0.481, R^2^ = 0.56, *p* = 4×10^−5^, red line: *y* = 0.245*x*+0.018, R^2^ = 0.73, *p* = 1×10^−5^; (C), 32 species (see Table S1, column E), blue line: *y* = 0.050*x*−0.194, R^2^ = 0.21, *p* = 0.017, red line: *y* = 0.154*x*−1.09, R^2^ = 0.82, *p* = 0.004; (D), 25 species (see Table S8), blue line: *y* = 0.072*x*−1.188, R^2^ = 0.81, *p* = 1×10^−4^; red line: *y* = 0.140*x*−2.370, R^2^ = 0.98, *p* = 3×10^−5^) for species with GI values of <1.5 (blue triangles) and >1.5 (red circles), respectively; mouse and human (when depicted) are indicated by green symbols. The inset in (B) shows the Akaike Information Criterion (AIC) values for models fitted with one to five linear slopes; note that a two-slope model best explains the data.

In order to test the bimodal distribution explicitly, we regressed GI values against neuroanatomical traits typically identified with (and studied in the field of) neocortical evolution: brain weight, neocortical volume, and neuron number. We found that each scaling relationship could be explained comparably well by either a non-linear function (Figure 3A) or two grade-shifted linear functions, with the best-fit linear models drawing significantly different slopes for high-GI and low-GI species (Figure 3B–3D). Specifically, by plotting GI as a function of cortical neuron number, we were able to determine, with two significantly different linear regressions for high- and low-GI species (T = 4.611, degree of freedom [d.f.] = 29, *p* = 2.8×10^−4^), demarcating values of 1±0.11×10^9^ neurons and 1.56±0.06 GI (Figure 3D), thus providing a neuron number correlate for the GI threshold. The deviation of these results from previous work, which have shown strong phylogenetic signals associated with both GI [31],[32] and neuron counts [33], may be explained both by our more than 2-fold increase in sampled species and the *a priori* assumption of previous work that GI and neuron number evolve as a function of phylogeny. Variation in GI, therefore, has not evolved linearly across the phylogeny, but has in fact been differentially evolved in two phenotypic groups.

### More Efficient Neurogenesis in Large-Brained Species

**Figure 4:**
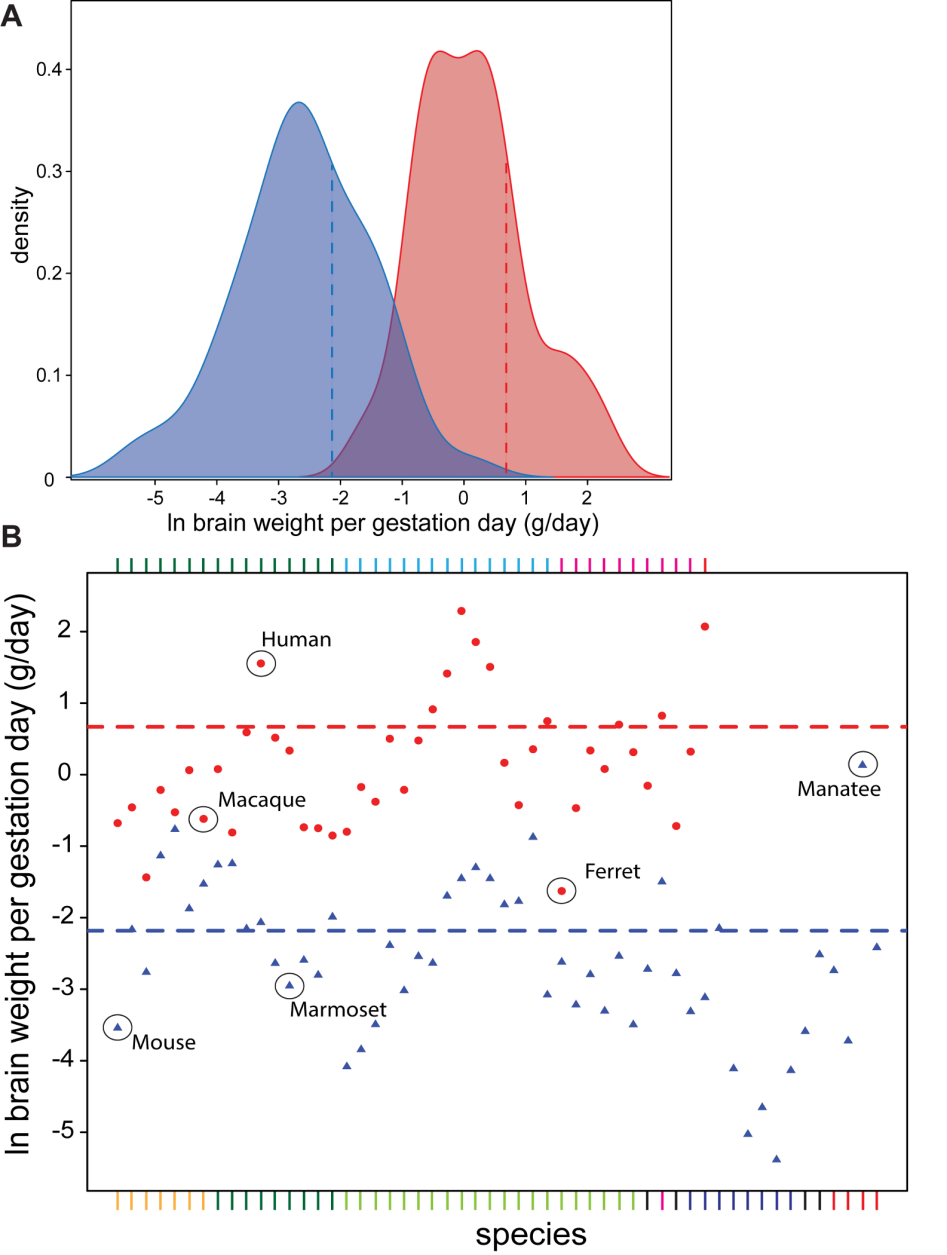
Brain weight generated per gestation day is considerably greater for high-GI than low-GI species. (A) Density plot showing the frequency of occurrence of the 96 eutherian species listed in Table S1 (omitting *C. volans*) with GI values of ≤1.5 (blue) and >1.5 (red) as a function of ln-transformed brain weight generated per gestation day. Note that the smallest values for brain weight per gestation day are found only in the low-GI group, while the largest values are found only in the high-GI group, but that the mean values for the two groups is also significantly different (dashed blue and red lines, T = 5.16, degree of freedom [d.f.] = 41, *p* = 4×10^−5^). (B) Ln-transformed plot of brain weight generated per gestation day for the 96 mammalian species (see A). Dashed blue line, mean value for GI≤1.5 (−2.04±0.047, standard deviation [SD]); red dashed line, mean value for GI>1.5 (0.583±0.050, SD). Selected organisms are indicated. The colors of the various high-GI and low-GI species shown above and below the plot, respectively, indicate the taxonomic groups as shown in Figure 1; the sequence of high-GI and low-GI species from left to right is according to Table S1, column A, top to bottom. Brain weight per gestation day was calculated from the data shown in Table S1, columns C and M.

By identifying an evolutionary threshold in the degree of gyrencephaly, as well as a correlate in terms of neuron number, we revealed the existence of two neocortical phenotypic groups, which found support in their distinct life-history associations (i.e., the GI is bimodally distributed and supports two principal mammalian phenotypes). These groups could be further divorced by accounting for the amount of brain weight accumulated per gestation day—a confident proxy for neonate brain weight per neurogenic period (Figure S6A and 6B)—which we show to be, on average, 14-times greater in high- compared to low-GI species (Figure 4). Notably, each GI group is constituted by both altricial and precocial species, so the degree of pre- versus post-natal development is not enough to explain the discrepancy in brain weight per gestation day in each group.

**Figure 5:**
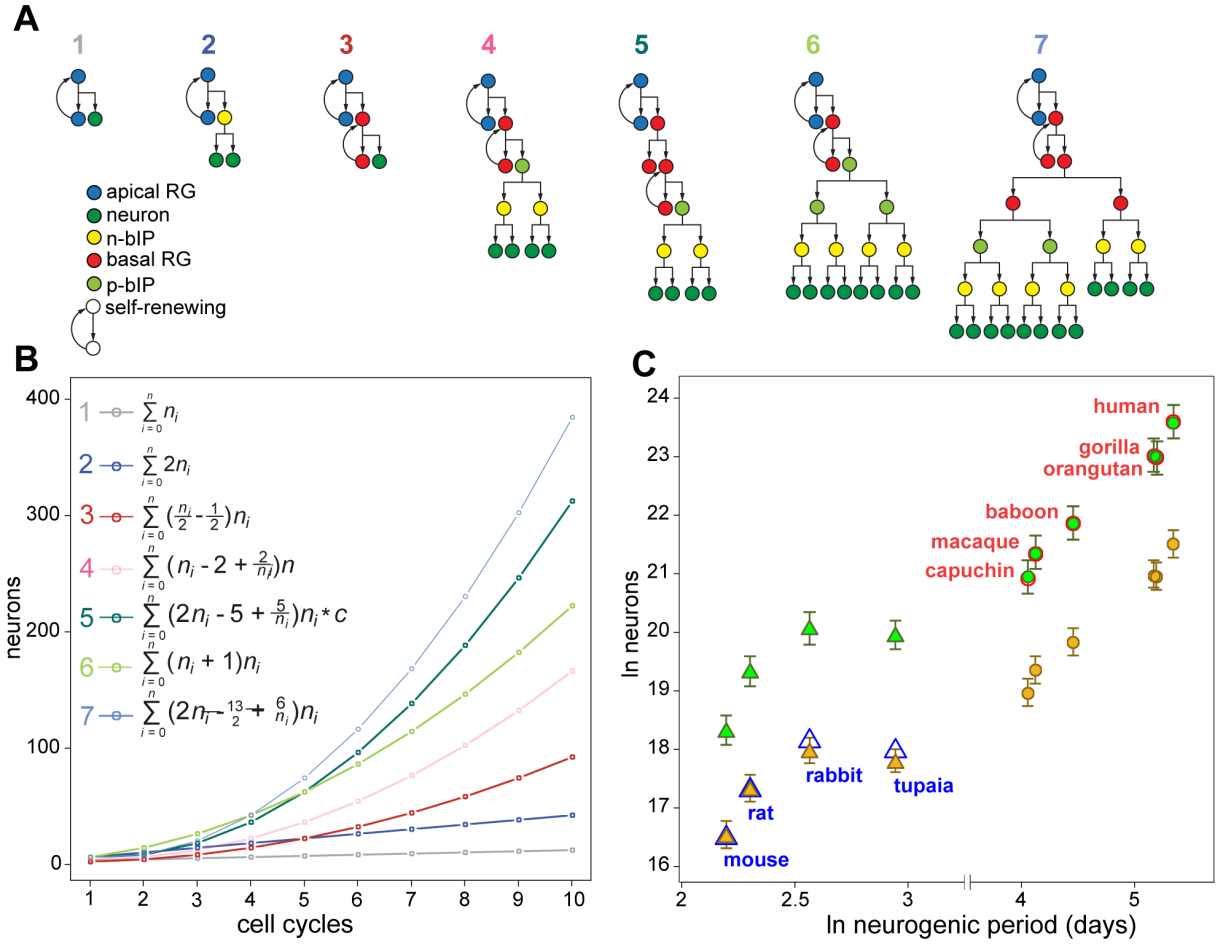
Distinct combinations of progenitor lineages are required to predict cortical neuron numbers for low- versus high-GI species. (A) Schematics of the seven lineages used to construct neuron output in species. Note that n-bIP refers to the neurogenic subtype of basal intermediate progenitors, and that p-bIP can be either the proliferative subtype of basal intermediate progenitors or bRG undergoing symmetric proliferative division [5],[10]. (B) Plotted neuron output of the lineages in (A), beginning with two apical RG cells, over ten cell cycles. Series to the left of the seven curves summarize the neuron output of each lineage, where *n*_i_ is the number of i divisions. A constant, c = 0.989, is incorporated into the series for lineage 5, allowing the series to converge on the true value of the lineage output as the number of divisions becomes increasingly numerous. (C) Ln-transformed plot of observed neuron counts as a function of neurogenic period for four species with a GI≤1.5 (open blue triangles) and six species with a GI>1.5 (open red circles). Predicted neuron counts were calculated using combinations of the lineages in (A), as specified in Table 2, that accurately fitted to the observed neuron counts either for mouse (closed gold symbols) or human (closed green symbols). Note that the mouse neurogenic program implicates only lineages 1–3, and the human neurogenic program only lineages 2–7. Errors bars represent 75% confidence intervals in cell-cycle length. See Figure S7 and Table S4 for observed and predicted data on a larger set of 17 species.

Rather, to explain the discrepancy, we introduced a deterministic model of cortical neurogenesis, using series summarizing seven neurogenic lineages (Figure 5A and 5B) and based on cell-cycle length, neuroepithelial founder pool size, neurogenic period, and estimates of relative progenitor-type population sizes (Tables 1 and 2). In total, 17 species were incorporated in the model, as we were limited by the number of species for which cortical neuron number was available. These species include species from four phylogenetic orders: Primata, Scandentia, Rodentia, and Didelphimorphia. We arrived at two models that show the highest reliability for predicting cortical neuron numbers in a range of species: a mouse neurogenic program, which implicates only asymmetrically dividing aRG and bRG cells and terminally dividing IPs (Figure 5A, lineages 1–3); and a human neurogenic program, which additionally implicates BPs undergoing symmetric proliferative divisions in the SVZ (Figure 5A, lineages 4–7). Each model is defined by the proportional occurrence of each lineage in that model (Table 2).

**Table 1:** Parameters for models of cortical neurogenesis.

Species	Gestation Period (d)	Neurogenic Period (d)^a^	Observed Neurons	Neuroepithelial Founder Pool (Cells)^a^	Cell-cycle Length (h)^b^
Human	270	112	1.63E+10	3.10E+07	45
Gorilla	257	103^c^	9.10E+09	1.59E+07	45
Orangutan	260	104^c^	8.90E+09	1.16E+07	45
Macaque	166	60	1.71E+09	4.41E+06	45
Baboon	180	72^c^	2.88E+09	6.37E+06	45
Capuchin	158	59^c^	1.14E+09	2.97E+06	45
Owl monkey	138	55^c^	4.42E+08	1.05E+06	30
Callimico	153	60^c^	3.57E+08	6.92E+05	30
Marmoset	146	58	2.45E+08	6.71E+05	30
Galago	134	54^c^	2.26E+08	1.01E+06	30
Tupaia	46	19^c^	6.04E+07	5.68E+05	18.5
Rabbit	30	13	7.15E+07	8.08E+05	18.5
Agouti	112	45^c^	1.10E+08	9.80E+05	18.5
Capybara	137	55^c^	3.10E+08	1.78E+06	18.5
Rat	21	10	3.10E+07	5.40E+05	18.5
Mouse	19	9	1.37E+07	3.99E+05	18.5
Opossum	NA	10	0.88E+06	2.50E+04	18.5

See Database S1.

a See Materials and Methods.

b See Materials and Methods and Figure S12.

c Estimate based on regression against gestation period (see Figure S6).

NA, not available.

**Table 2:** Best-fit proportional occurrences (%) of lineages in different taxa.

Taxa	Lineages (%)						
	1	2	3	4	5	6	7
Human^a^	0	20	40	10	10	10	10
Gorilla	0	20	40	10	10	10	10
Orangutan	0	20	40	10	10	10	10
Baboon	0	20	40	10	10	10	10
Macaque^a^	0	20	40	10	10	10	10
Capuchin	0	20	40	10	10	10	10
Owl monkey	0	50	50	0	0	0	0
Callimico	0	50	50	0	0	0	0
Marmoset^a^	0	60	40	0	0	0	0
Galago	0	75	25	0	0	0	0
Tupaia	10	75	15	0	0	0	0
Rabbit^a^	10	75	15	0	0	0	0
Agouti	10	75	15	0	0	0	0
Capybara	10	75	15	0	0	0	0
Rat	10	80	10	0	0	0	0
Mouse^a^	10	80	10	0	0	0	0
Opossum	80	10	10	0	0	0	0

See Figure 5.

a Supported by observational data (see Materials and Methods).

Using the mouse neurogenic program we were able to predict neuron counts within 2% of the observed counts for mouse and rat, but underestimated neuron counts by more than 80% in high-GI species (Figure 5C; Table S4). Increased proportional occurrences of the bRG lineage 3 (Figure 5A) with increasing brain size was required to achieve estimates with <5% deviation from observed neuron counts in the other low-GI species (Table 2; Figure S7). The human neurogenic program predicted neuron counts within 5% for all six high-GI species, but overestimated neuron counts by more than 150% for the low-GI species. Estimates of proportional occurrences of the various lineages in the mouse, marmoset, rabbit, macaque, and human are supported by previous work detailing relative abundances of different progenitor cell-types during cortical neurogenesis [7],[9]–[11],[14],[22] (IK and WBH, in preparation). Evolutionary gain or loss of proliferative potential in the SVZ is an essential mechanistic determinant of neocortical expansion, such that the presence of symmetric proliferative BP divisions in high-GI species and their absence in low-GI species is sufficient and even requisite for explaining neocortical evolution (Figure S8). Notably, the lissencephalic opossum, a marsupial species with extreme altriciality, required a decreased proportional occurrence of the bIP-containing lineage 2 (Figure 5A) and an increased proportional occurrence of the direct neurogenic lineage 1 (Figure 5A) but, like all species analyzed here, could not achieve its observed neuron count without the bRG-containing lineage 3 (Figure 5A). This suggests that bRG cells are ancestral at least to the therian stem [34].

### Adaptive Evolution of Proliferative Potential in the SVZ

**Figure 6:**
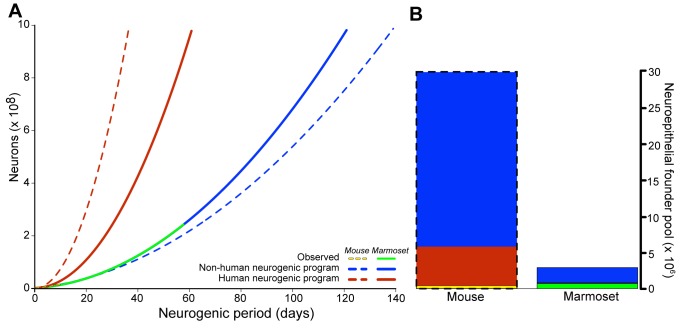
Mouse and marmoset, both low-GI species, may generate 10^9^ neurons more efficiently by adopting the human neurogenic program than by extending neurogenic period or expanding neuroepithelial founder pool size. (A) Using its observed neurogenic program (yellow dashed line), the mouse may achieve 10^9^ neurons by extending its observed neurogenic period 14-fold (blue dashed line) or, by using the human neurogenic program (red dashed line), 4-fold. Similarly, the marmoset (green solid line) may achieve 10^9^ neurons using the human neurogenic program (solid red line) in 50% of the time it would take using its observed neurogenic program (solid blue line). (B) The barplot shows the amount by which both species' neuroepithelial founder pools would have to increase to achieve 10^9^ neurons using either their observed (blue) or the human (red) neurogenic program. In (A) and (B), yellow and green line endpoints (A) and bar heights (B) represent observed values for mouse and marmoset, respectively. See Table S4 for primary data and estimates.

To simulate the adaptiveness of evolving increased proliferative potential in the SVZ in two lissencephalic species—mouse and marmoset—we calculated trade-offs between neuroepithelial founder pool size and neurogenic period using mouse/marmoset and human programs of cortical neurogenesis to achieve 10^9^ neurons. We show that, in both species, evolving a lineage of BPs capable of symmetric proliferative divisions is between two and six times more cost-efficient than either expanding founder pool size or lengthening neurogenesis; and that the marmoset, by evolving such proliferative BPs, could achieve 10^9^ neurons by increasing either its observed founder pool or neurogenic period less than 15% (Figure 6).

We further clarified the significance to neuron output of each progenitor-type with deterministic and stochastic models of temporal dynamics and progenitor cell-type variables. The proportional contributions of each lineage to overall neuron output in the mouse and human neurogenic programs were calculated using stage-structure Lefkovitch matrices. By excluding lineages one at a time, we determined the degree to which each lineage contributed to total neuron production. From these analyses, it was clear that symmetric proliferative BPs are increasingly necessary in larger brains and that any exponential increase in neuron production is statistically implausible in the absence of such BPs (Table S5).

Finally, we described the dynamics of asymmetric self-renewing versus symmetric proliferative progenitors, isolated from their observed lineage beginning at the apical (ventricular) surface, by introducing three ordinary differential equations (ODEs) modeling a self-renewing cell that generates either a differentiated cell or proliferative cell. The ODEs describe a self-renewing mother progenitor, which can generate either a post-mitotic neuron or a proliferative daughter at each division. The proliferative daughter is allowed one symmetric proliferative division followed by self-consumption. The likelihood of a neuron or proliferative daughter being generated by the mother, therefore, is interdependent. We also include the pool of mother progenitors as a linear variable. We show that neuron output of the system increases dramatically when both the initial pool of self-renewing cells and the likelihood of those initial cells to generate proliferative, rather than differentiated, cells approaches saturation (Figure S9).

## Discussion

The emergence of new structures, in the most general sense, is typically limited to selection on existing developmental processes; and conserved pathways may persist, over evolutionary time, even when the phenotype is transformed or unexpressed [35]–[37]. However, it is also evident that development may be adapted without affecting phenotype (e.g., [38],[39]). Therefore, in order to understand selective pressures acting on a discontinuous or convergent trait, it is necessary to investigate the underlying developmental processes generating it.

### The Evolution of Two Principal Mammalian Phenotypes

We have shown that a gyrencephalic neocortex is ancestral to mammals. This finding is concordant with evidence [29] that the mammalian ancestor was relatively large (>1 kg) and long-lived (>25-year lifespan) and, furthermore, provides considerable resolution to recent evidence for a gyrencephalic eutherian ancestor [28] by sampling nearly twice as many species and categorizing gyrencephaly as a continuous, rather than a binary, trait.

More surprisingly, we show that convergent evolution of higher orders of gyrencephaly along divergent lineages has been accompanied by two distinct constellations of physiological and life-history paradigms. Specifically, species with a GI>1.5, which is commensurate with 1 billion cortical neurons, exhibit patterns of development and life-history that are distinct from species with a GI≤1.5, irrespective of phylogeny. This implies that there is a considerable constraint on either the ability of species of a given neocortical size to exploit certain ecologies or the potential for species of a given ecology to freely adapt neocortical size. Even marine mammals, whose selection pressures are sui generis, may largely be held to the same evolutionary stereotyping as terrestrial mammals (Figure S10).

While our results countenance previous studies showing associations between physiological and life-history traits in mammals (see [40]), we identify those traits to have a bimodal distribution, rather than to vary allometrically, across species. This distribution depicts a Waddington-type landscape for neocortical expansion—albeit relevant at the species-level—wherein the GI threshold represents an adaptive peak requiring a particular adaptation in neurogenic programming within a population for traversal. Our results may explain this landscape by mechanistic differences occurring during cortical neurogenesis between species above and below the GI threshold: the necessity of symmetric proliferative BPs in high-GI species and their putative absence in low-GI species.

### Neurogenic Period, Not Programming, Is Sufficient to Differentiate High-GI Species

The human neurogenic program constructed here clearly shows that the same neurogenic lineages in the same proportions are required to generate the neocortices of Old World monkeys, apes, and humans, and may even be extended to carnivores, cetartiodactlys, and other high-GI species (Figure S10), demonstrating that neurogenic period alone may be sufficient to explain differences in neocortical size between any species in the same GI group (Figure S11). Our data are insufficient, however, to determine whether these adult differences are uniform across the neocortex or differentially represented in infra- versus supra-granular layers [20],[41].

We propose that symmetric proliferative divisions of BPs, in addition to having an abundance of bRGs in an expanded SVZ, are necessary and sufficient for the evolution of an expanded and highly folded neocortex in mammals. Recent work in the fetal macaque supports this proposal [10]. We thus conclude that an increase in the proliferative potential of BPs is an adaptive requirement for traversing the evolutionary GI threshold identified here. But because we reconstruct the eutherian ancestor to have a GI value of 1.48±0.13 (standard error of the mean [SEM]) (Table S3), which falls within the range of the observed threshold, we are left with an ambivalent evolutionary history for mammalian neocortical expansion: either (i) BPs capable of undergoing symmetric proliferative divisions are ancestral to all eutherian mammals and were selected against along multiple lineages (e.g., rodents, strepsirrhines), so that the ultimate loss of BP proliferative potential in certain taxa, and therefore the evolution of low-GI species, is the result of divergent developmental adaptations; or (ii) such symmetric proliferative BPs are not ancestral to eutherian mammals, but evolved convergently along multiple lineages, in which case the developmental process for their inclusion in neurogenic programming may be conserved, even if that process was unexpressed for long stretches of mammalian evolution.

### Conclusion

We have revealed an important insight into mammalian evolution: a GI threshold exists in mammalian brain evolution; neocortical expansion beyond that threshold requires a specific class of progenitor cell-type (BPs) to adopt a specific mode of cell division (symmetric proliferative); and the difference in neuron output between any species on the same side of that threshold does not appear to require adaptations to the lineage or mode of cell division during neurogenesis, but may simply reflect differences in the length of the neurogenic period. Further research into the conservation of genomic regions regulating the capacity of BPs to undergo symmetric proliferative divisions (e.g., through the establishment and maintenance of a proliferative niche in the SVZ) in low- versus high-GI species may reveal whether this mechanism for neocortical expansion has evolved independently in distantly related species or is the product of a deep homology in mammalian cortical development.

## Materials and Methods

### Calculating GI

We calculated GI using images of Nissl-stained coronal sections from http://brainmuseum.org. We used 10–22 sections, equally spaced along the anterior-posterior axis of the brain, for each species (Figure S1). The inner and outer contours of the left hemisphere were traced in Fiji (http://fiji.sc/wiki/index.php/Fiji). The species for which we calculated GI are indicated by an asterisk in Table S1. Additional GI values were collected from the literature (Table S1; Database S1). Several species (e.g., platypus), whose cortical folding has been described [42],[43] but not measured according to the method established by [44], could not be included in our primary reconstructions of GI evolution (Figure 1). However, these species, assumed to be lissencephalic (GI = 1.0), were included in supplemental analyses (Table S3; see Reconstructing the evolutionary history of GI).

Work in humans and baboons has shown that interindividual variation in GI is not enough to outweigh interspecific differences [45],[46].

### Reconstructing the Evolutionary History of GI

Variation in the mode and tempo of a continuous character trait is not always best characterized by a random walk (i.e., Brownian motion). Therefore, we compared a range of evolutionary models on the phylogenetic distribution of GI to find the best fit for the data [47]–[50]. Log-likelihood scores for each model were tried against the random walk score using the cumulative distribution function of the χ^2^ distribution. Maximum-likelihood ancestral character states of GI and rate-shifts in the evolution of GI were then constructed using the best-fit model, with the standard error and confidence intervals calculated from root node reconstruction in PDAP using independent contrasts [51]–[53]. Although a number of putatively lissencephalic non-eutherians were unavailable for our analyses (see Calculating GI), we nonetheless reconstructed alternative ancestral GI values that included one hypothetical monotreme and three hypothetical marsupials (Table S3). The phylogeny used in this analysis was derived from a species-level supertree [26]. We appreciate that the phylogenetic hypothesis reconstructed by [54] gives notably deeper divergence dates for mammalian sub-classes; however, not enough of our sampled species were included in this reconstruction for it to be useful here.

To trace evolutionary changes in GI at individual nodes and along lineages, we used a two-rate mode that highlighted the differences in high (>1) versus low (<1) root-to-tip substitutions and then sampled rates based on posterior probabilities across the tree using a Monte Carlo Markov Chain. We assumed that transitioning between adjacent GI values had the highest likelihood of occurrence. The rate at a given node could then be compared to the rate at the subsequent node to determine if a rate transition was likely. We corroborated these results using the auteur package [55], which calculates rate-transitions at internal nodes under the assumption of an Ornstein-Uhlenbeck selection model [34] over 1 million Monte Carlo sampling iterations drawn from random samplings of posterior distributions of lineage-specific rates.

Scaling relationships were determined for GI as a function of all continuous life-history and physiological traits, including adult cortical neuron counts. For three eulipotyphla species (*Sorex fumeus*, *Blarina brevicauda*, *Scalopus aquaticus*), data were available for neuron counts but not GI, and therefore we extrapolated the GI of those species on the basis of gross morphology. Finally, to test whether the bimodal distribution of GI may be influenced by the topology of the mammalian phylogenetic tree, we used an expectation-maximization algorithm. Each simulated trait was given the same variance as GI (Figure S5) and the result was averaged over 10^4^ simulated datasets. None of the simulations produced the same bimodal distribution of species observed for GI data.

### Stochastic Mapping of GI across the Mammalian Phylogeny

We used a comprehensive phylogenetic approach to map 37 life-history and physiological character traits collected from the literature (Tables S1 and S2) onto hypotheses of phylogenetic relationships in Mammalia, in order to examine how those traits correlate, over evolutionary time, with degree of gyrencephaly. Continuous character traits were discretized using the consensus of natural distribution breaks calculated with a Jenks-Caspall algorithm [56], model-based clustering according to the Schwarz criterion [57], and hierarchical clustering [58]. Character histories were then corrected for body mass with a phylogenetic size correction [59],[60] and summarized across the phylogeny using posterior probabilities. Associations between individual states of each character trait along those phylogenetic histories were calculated in SIMMAP (v1.5) using empirical priors based on the frequency of character states for each trait [61]; the association between any two states was a measure of the frequency of occurrence (i.e., the amount of branch length across the tree) of those states on the phylogeny. While correcting for body mass is intended to normalize the data, it cannot completely remove interdependencies between character traits. Although we cannot *a priori* assume that any of the traits interact, exploring interactions between them deserves further investigation.

The sums, rates, and types of changes for GI and body weight were plotted as mutational maps to assess directional biases in their evolution [62]–[64]. These were used to determine the evolutionary historical patterns of GI and, as a control, body weight. By estimating the occurrence (number of times an increase/decrease happens) and timing (where in the phylogeny the change occurs) of different values for each trait, we were able to calculate how often each trait has increased and decreased in mammalian evolution. We were therefore able to evaluate the ratio of increases over decreases for each trait (Figure S4).

### Estimating Neuroepithelial Founder Pool Populations

We estimated neuroepithelial founder pool populations for mouse and human. For the mouse, we used coronal sections of an E11.5 mouse embryo obtained from the Allen Brain Atlas [65]. We obtained 19 sections equidistantly spaced along the anterior-posterior axis of the brain. The length of the ventricular surface of the dorsal telencephalon was manually traced in Fiji [66] on each section starting from the point above the nascent hippocampus and ending in the point above the lateral ganglionic eminence. The horizontal length of the embryonic brain at E11.5 was measured with images from [67]. Using the coronal and horizontal measurements, we constructed a polygon representing the ventricular surface of the dorsal telencephalon and calculated the area of this surface in Fiji. We measured the surface area of the end-feet of neuroepithelial cells using EM images of the coronally cut apical surface of an E11.5 embryonic mouse brain (Table S6). The diameter of a single end-foot was calculated by measuring the distance between the adherens junctions. We corroborated these end-feet calculations with published immunofluorescence stainings of the apical complex (ZO1 and N-cadherin) from an *en face* perspective [68],[69]. The average surface area of a single end-foot was calculated by approximating the end-foot as a hexagon; and the number of founder cells was estimated by dividing the surface of the dorsal telencephalon by the surface of an individual end-foot of the neuroepithelial cell, such that
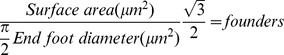
Our final mouse values were comparable to those previously published [70]. For the human, we followed the same procedure, using ten coronal sections and one horizontal section of a gestation week (GW) 9 brain [71]. End-feet were calculated using EM images of the apical surface of a human brain at GW13. The measurements are available in Table S6. Because the number of founder cells per surface area was nearly equivalent in mouse and human (4×10^5^/mm^2^), we used this ratio, along with data on ventricular volume collected from the literature (Tables S1 and S2; Database S1), to estimate neuroepithelial founder cell populations for a further 15 species (Table 1). For species where no data on ventricular volume were available, values were estimated on the basis of a regression analysis against brain weight (Figure S6). Ventricular volume was then converted to surface area for each species by approximating the ventricle as a cylinder with a 4.5-to-1 height-to-diameter proportion (this ratio was estimated on the basis of observations in mouse). Ventricular volume-derived ventricular surface area estimates were corroborated with the surface areas calculated from the literature for mouse and human. Founder cell estimates were then computed on the basis of the densities derived above for mouse and human. Using this method, but alternately ignoring our mouse and human calculations to define the parameters, we were able to predict mouse and human values within 10% of our calculations, respectively.

### Mathematical Modeling of Neurogenesis

Workers have demonstrated the occurrence of three primary lineages of neuron generation in mouse corticogenesis (Figure 5A, lineages 1–3) [1],[5],[14],[72] and a further four lineages in primate corticogenesis (Figure 5A, lineages 4–7) [9],[10]. While there is evidence for at least one additional lineage in mouse [6], and further lineages may be speculated, we limited our model to the seven that are considered to contribute most significantly to neuron output [2],[10],[73],[74]. The extent of neuron generation in each of these seven lineages was summarized in series and solved numerically (Figure 5B).

Neurogenic period was either taken from the literature (Database S1) or estimated on the basis of a regression analysis of neurogenic period as a function of gestation period (Figure S6). Neurogenic period in human was estimated using empirical observations from the literature [75]–[77]. The averaged cell-cycle length for apical and BPs from the mouse (18.5 hours) [78]–[80] was used for all non-primates; averaged cell-cycle length for cortical areas 17 and 18 from the macaque (45 hours) was used for catarrhines [10],[81]; and an intermediary cell-cycle length (30 hours in marmoset, determined by EdU labeling; Ayako Murayama and Hideyuki Okano, personal communication), was used for platyrrhines. Using an average cell-cycle length value for all progenitor-types was found to be equally valid for predicting neuron number as using different cell-cycle lengths for each progenitor-type (Figure S12). Therefore, despite its potential shortcomings, using average cell-cycle length is a valid approach and, given the scarcity of species data on the cell-cycle length of various progenitor-types at different stages of neurogenesis, the best approach available to construct neurogenic models across many species. Generous confidence intervals (75%) for cell-cycle length are used in our models (Figure 5C), in order to show the minimal explanatory power cell-cycle length provides for interspecific differences in cortical neuron number.

Diminishing numbers of neuroepithelial cells have been observed to continue to proliferate at the ventricle until E18.5 in the mouse [7]. Therefore, final neuroepithelial founder pool estimates were calculated from the aforementioned by evenly decreasing the value of *n* in the Sherley equation [82] from 1 at E9.5 to 0 at E18.5 in the mouse and at comparable neurogenic stages in other species.

Neuron numbers were calculated for each species from combinations of lineages. The proportional contribution of each lineage for each species was parameterized according to existing data on progenitor cell-type abundances in mouse [14], marmoset [22], rabbit (IK and WBH, in preparation), macaque [10], and human [9],[11]. Where no such data were available, proportional contributions were permutated for all lineages until a best-fit estimate, based on cortical neuron numbers taken from the literature [33],[83]–[85], was achieved (Tables 1 and 2). Each lineage was assumed to occur from the first to final day of neurogenesis, although this is only approximately accurate. Finally, because of published estimates of postnatal apoptosis in the mammalian cortex [86]–[88], we assumed neuron counts to be 1.5-fold higher at the termination of neurogenesis than in the adult brain; therefore, neuron number at the termination of neurogenesis was estimated in each species by multiplying neuron numbers collected from the literature by 1.5. This multiplication is not represented in Table 1.

### Calculating the Effects of Proliferative Progenitors on Neuronal Output

Trade-offs in adapting a human neurogenic program with either an expanding neuroepithelial founder pool or lengthening neurogenic period were tested for the mouse (*Mus musculus*) and marmoset (*Callithrix jacchus*), two lissencephalic species whose cell-type proportions during neurogenesis have been documented [7],[14],[22].

To estimate the relative reproductive value and stable-stage proportions of each of the lineages in the mouse and human neurogenic programs, we constructed a stage-structured Lefkovitch matrix, using sums of the lineage series (after 100 cycles) as fecundity values and complete permutations of the proportional contributions of each lineage as mortality values. The altered growth-rates of each lineage were calculated by excluding lineages one at a time and assuming 100% survival in the remaining lineages (Table S5).

We introduced three ODEs to explore the average dynamics of asymmetric versus symmetric progenitors, such that: if a(t), b(t), and c(t) are the numbers of asymmetrically dividing cells, differentiated cells, and proliferative cells, respectively, then,
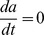


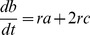


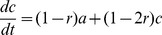
where *r* is equal to growth-rate. If *a(t)* = *a_0_*, then
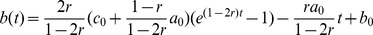
and
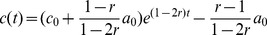
Using these ODEs, we calculated the effect on neuron output of increasing the likelihood of symmetrically dividing daughter progenitors in the lineage (Figure S9). The interdependent growth-rates in the model reflect a purely mechanistic interpretation of determining neuronal output from a finite pool of asymmetrically dividing cells. The ODEs, therefore, may not reflect differential regulation of neuronal output via direct versus indirect neurogenesis. The daughter proliferative cells are designed to carry out one round of proliferation followed by a final round of self-consumption (Figure S9).

## Supporting Information

Figure S1 **Determination of GI.** Coronal section of the brain of an adult house cat (*Felis catus*) (obtained from www.brainmuseum.org) illustrating the method used to calculate GI values as described in [44]. Green line, actual contour; magenta line, hypothetical outer contour.
(TIF)

Figure S2 **Maximum-likelihood ancestral node reconstruction of GI values at all internal nodes of the mammalian phylogeny.** Reconstruction based on a delta (δ = 2.635) selection model. Barplot shows the distribution of GI values across the phylogeny; dashed red line indicates GI = 1.5. See Table S1, column F, for GI values.
(TIF)

Figure S3 **Rate-transitions in the mutation rate of GI values along lineages of the mammalian phylogeny.** (A) A two-mode selection model that weights low over high root-to-tip substitutions. Numbers on the branches indicate the change in mutation-rate compared to the previous branch: 0 values indicate no significant change; values >0 indicate significant change (*p*<0.05). Note the especially high rate-transitions leading to primates, cetartiodactyls, and cetaceans (open blue circles). (B) Mutation- and transition-rate estimates of GI values using an Ornstein-Uhlenbeck selection model. Branches are colored to illustrate whether the mutation-rate estimates along each lineage are above (red) or below (blue) the median rate (orange); nodes are circled to indicate the posterior support of a transition-rate-shift event. The gradient of colors (see key) indicates the degree of deviation of the mutation-rate estimates (branches) and transition-rate estimates (nodes) from the median, with the highest deviation being arbitrarily set to ±1.0 and the median to 0.0; the size of the circles (see key) at the nodes indicates the degree of posterior support for a transition-rate-shift event, with the highest value being arbitrarily set to 1.0 and lack of support to 0.0. Note that simians have evolved GI values at a rate consistent with the mammalian median. See Table S1, column F, for GI values.
(TIF)

Figure S4 **Barplots of types of transitions over mammalian evolution between four GI groups (see** **Figure 2A** **) and between five body-mass groups (see Tables S1 and S2) averaged over 10^5^ simulations.** The number of total transitions from one GI (left) or body mass (right) group to another is summed as either high-to-low or low-to-high transitions. Note that significantly more high-to-low than low-to-high transitions are observed for GI, but that no significant difference in type of transition is observed for body mass. Error bars, standard error of the mean (SEM).
(TIF)

Figure S5 **The bimodal distribution of GI values across the mammalian phylogeny is non-random.** A histogram showing the frequency of occurrence of GI values, binned at 0.1 intervals, for the 102 mammalian species listed in Table S1. Blue, GI values ≤1.5; red, GI values >1.5. The bimodal distribution of GI values shows a natural break at GI = 1.5, which is supported by energy-based hierarchical clustering (see Figure 2B). Note the possibility for a third GI group (GI>3, yellow), constituting cetaceans and elephant; however, we have too few sampled species from these orders to assess the group decisively (see Figure S10). See Table S1, column F, for GI values.
(TIF)

Figure S6 **Ln-transformed plots of neonate brain weight (A) and ventricular volume (B) as functions of adult brain weight; neurogenic period as a function of gestation period (C); and a plot of neuroepithelial founder cells as a function of ventricular surface area (C).** (A) Neonate brain weight scales linearly with adult brain weight for 52 eutherian species (*y* = 1.09*x*−1.49, R^2^ = 0.92, *p* = 6×10^−7^). (B) Ventricular volume scales linearly with adult brain weight for 30 eutherian species (*y* = 0.93*x*+2.37, R^2^ = 0.93, *p* = 9×10^−8^). (C) Neurogenic period scales linearly with gestation period for a sample of seven species (*y* = 0.91*x*−0.42, R^2^ = 0.94, *p* = 0.0002), spanning two mammalian superorders. (D) Ventricular surface area, converted from ventricular volume (see Estimating neuroepithelial founder pool populations), scales linearly with our estimated neuroepithelial founder populations (*y* = 6.7×10^5^+878*x*, R^2^ = 0.94, *p* = 5×10^−8^). (A, C) Note that these plots demonstrate the strong predictive powers of adult brain weight and gestation period for neonate brain weight and neurogenic period, respectively, validating the assumptions made in Figure 4. Mouse and human are indicated by filled green circles. See Table S1 for primary data.
(TIF)

Figure S7 **Stacked barplot, for the indicated species, of deviations between the observed cortical neuron counts and those predicted based on human (red), mouse (blue), and marmoset (yellow) neurogenic programs (see** **Figure 5** **and** **Table 2** **).** For each species, deviations were calculated as |100 * ((Predicted−*Observed)/Observed)*| and then divided by the sum of deviations obtained for all three programs. Predictions based on the marmoset program deviate from observed neuron counts considerably for the six species with a GI value >1.5 (red text), but also slightly for species with a GI≤1.5 (blue text), indicating a necessity for differential proportional occurrences of bRG in low-GI species. It is worth noting that natural intraspecific variation in cortical neuron number has been shown to be considerably less than interspecific variation [89],[90]. See Table S8 for primary data.
(TIF)

Figure S8 **Plot of observed cortical neuron number (red circles) as a function of neurogenic period for six species with a GI value >1.5.** Predicted neuron numbers are presented for the human neurogenic program (green circles; see Figure 5, Table 2) and for two further lineages, each of which is assumed to have a 100% proportional occurrence: direct neurogenesis from bRG (blue circles) and indirect neurogenesis from bRG via a self-consuming bIP cell (orange circles). Note that indirect neurogenesis from bRG via n-bIP is nearly sufficient to achieve the observed neuron count in the Capuchin monkey, but not that of human. See Tables S1 and S8 for primary data.
(TIF)

Figure S9 **Neuron outputs from solutions to ODEs describing direct versus indirect neurogenesis for growth-rate values ≤0.5.** Contour plot of neuron densities, *b(t)*, for a varying initial asymmetrically dividing cell population, a, and likelihood of direct (r = 1) versus indirect (r = 0) neurogenesis. Note that neuron output increases maximally when both the initial cell pool increases (a→100) and the likelihood of indirect neurogenesis increases (r→0). Here, *b(0)* = 2, *c(0)* = 2, and *t* = 10. The optimal *r* to maximize *b(t)* approaches 0.5*t* when *t* is fixed.
(TIF)

Figure S10 **Neocortical development in marine mammals may be largely explained by the same neurogenic program as terrestrial mammals.** (Top) Observed cortical neuron numbers for human, four cetacean species, and one marine carnivore (Harp seal, *Pagophilus groenlandicus*) are shown beside neuron numbers calculated from the human (red) and mouse (blue) neurogenic programs. Asterisks denote neuron numbers that are significantly different (T>7, *p*<0.05) from the observed; error bars are 95% confidence intervals. Note that the Bottlenose dolphin (*Tursiops truncatus*) is the only species for which the human program is not sufficient to achieve its observed number of neurons. (Bottom) The number of neurons generated per neurogenic day (green) and per body weight (gold) in human and the five marine mammals. Although the fin whale generates more neurons per neurogenic day, the human program produces a higher neuron count due to the fin whale's large estimated founder pool. See Tables S1 and S7 for primary data.
(TIF)

Figure S11 **Neocortical complexity, represented here as cortical gyrification, is tightly linked to progenitor behavior in the SVZ.** The nature of the link is such that incremental changes to SVZ progenitor behavior (inner ring) may effect exponential changes in neocortical complexity (outer ring). Therefore, only minor changes in the proliferative capacity of BPs (yellow arrow, inner ring) are needed to distinguish the major differences in neocortical complexity (yellow arrow, outer ring) between the macaque and human. It remains to be shown whether shifts in the proliferative capacity of BPs and neocortical complexity can occur independently (i.e., whether the black arrows can be bent). Pictured clockwise: mouse, capybara, ferret, macaque, human.
(TIF)

Figure S12 **Cell-cycle length of cortical progenitors and neuron number predictions in non-primates.** (A) Cell-cycle length of mouse *Tis21*-GFP–positive (+) and –negative (−) apical (AP) and basal (BP) progenitors at different stages of neurogenesis. Values for AP(−) and AP(+) at embryonic day (E) 10.5 (onset of mouse cortical neurogenesis) are taken from [79], values for AP(−), AP(+), BP(−), and BP(+) at E14.5 (mid-neurogenesis) are from [80], and the other values are extrapolated considering the data of [78]. (B) Barplot of the observed number of neurons (grey columns) in the cortex of four rodents, the rabbit, and *Tupaia* (a sister species to primates) compared to the number of neurons predicted using a fixed cell-cycle length value of 18.5 hours (blue columns, Table 1, i.e., the averaged cell-cycle length for apical and BPs from the mouse), as was done in Figure 5C, and the number of neurons predicted using dynamic cell-cycle length values for each progenitor class as shown in (A) (yellow columns). Note that for all species the predictions based on fixed and dynamic cell-cycle length values deviate from each other by <1%. The percentage deviations between observed and mouse neurogenic program-predicted neuron numbers are listed in Table S4.
(TIF)

Table S1 **Physiological and life-history variables.** ¶See Table S2 for column definitions and Database S1 for data. *Calculated from images of Nissl-stained coronal sections from www.brainmuseum.org (see Materials and Methods).
(XLSX)

Table S2 **Definitions of physiological and life-history variables.**
(XLSX)

Table S3 **Ancestral GI reconstructions including hypothetical lissencephalic marsupials and monotreme.**
(XLSX)

Table S4 **Predicted neuronal counts based on human, mouse, and marmoset programs.**
(XLSX)

Table S5 **Stable-stage distribution and reproductive values for phase-structured neurogenic programs.** *See Figure 5.
(XLSX)

Table S6 **Parameters for neuroepithelial founder pool population estimates in mouse and human.**
(XLSX)

Table S7 **Neocortical neuron number predictions in marine mammals based on human and mouse neurogenic programs.** *Estimated from neuron density; **estimated from gestation period (see Materials and Methods).
(XLSX)

Table S8 **Neocortical neuron number for** **Figure 3D** **.**
(XLSX)

Database S1 **The GI and life-history trait database (102 species) was assembled from the literature (see references 1–15 in Database S1).**
(DOC)

## Abbreviations

aRG: apical radial glia
bIP: basal intermediate progenitor cell
BP: basal progenitor
bRG: basal radial glia
GI: gyrencephaly index
ODE: ordinary differential equation
SVZ: subventricular zone
VZ: ventricular zone
