# Supplementary figures and images for: An Adaptive Threshold in Mammalian Neocortical Evolution
Source: PLoS Biol. 2014 Nov 18;12(11):e1002000. doi: 10.1371/journal.pbio.1002000 (PMC4236020; doi:10.1371/journal.pbio.1002000)

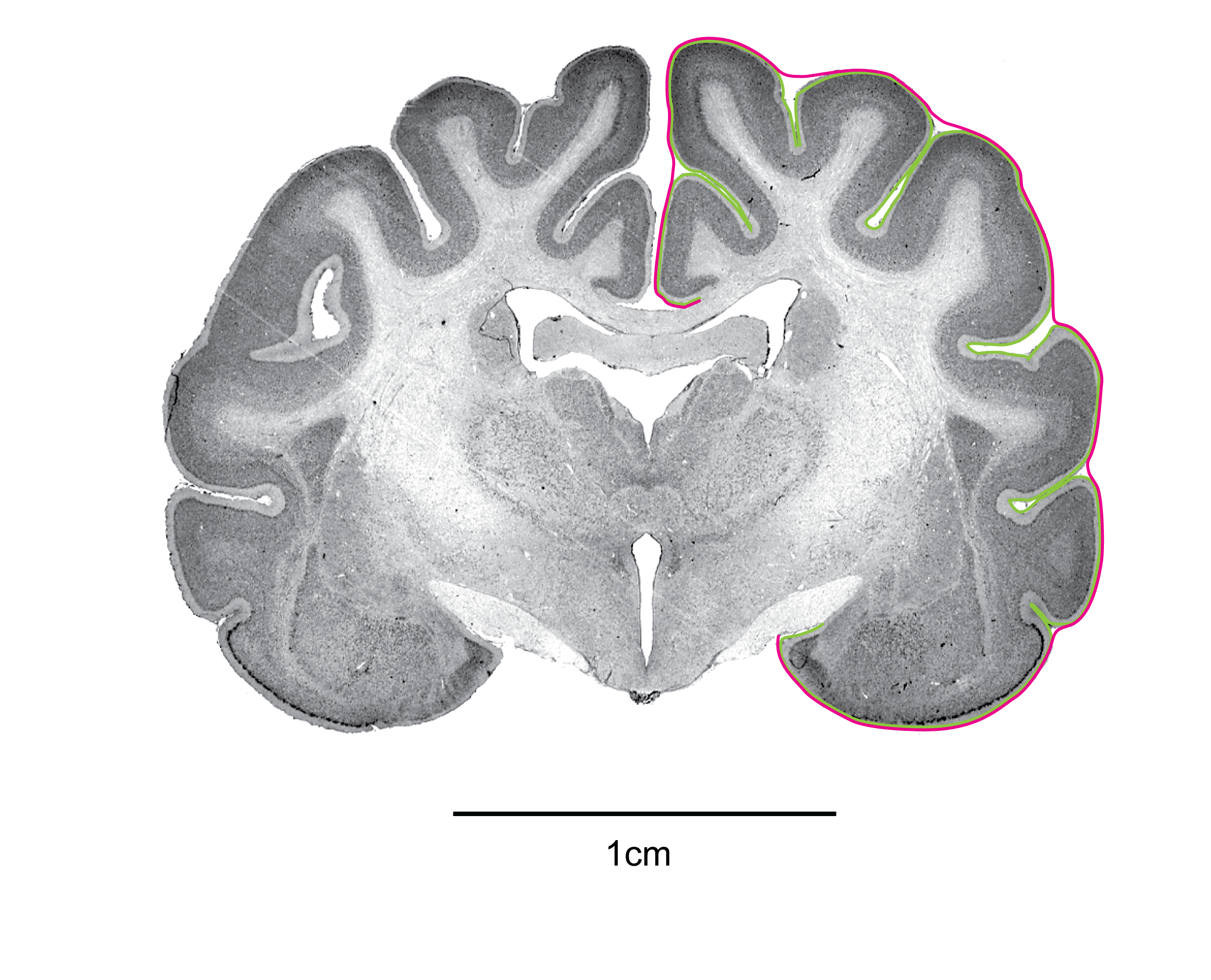

Supplement: Figure S1 — Determination of GI. Coronal section of the brain of an adult house cat (Felis catus) (obtained from www.brainmuseum.org) illustrating the method used to calculate GI values as described in [44]. Green line, actual contour; magenta line, hypothetical outer contour. (TIF) [file pbio.1002000.s001.tif]

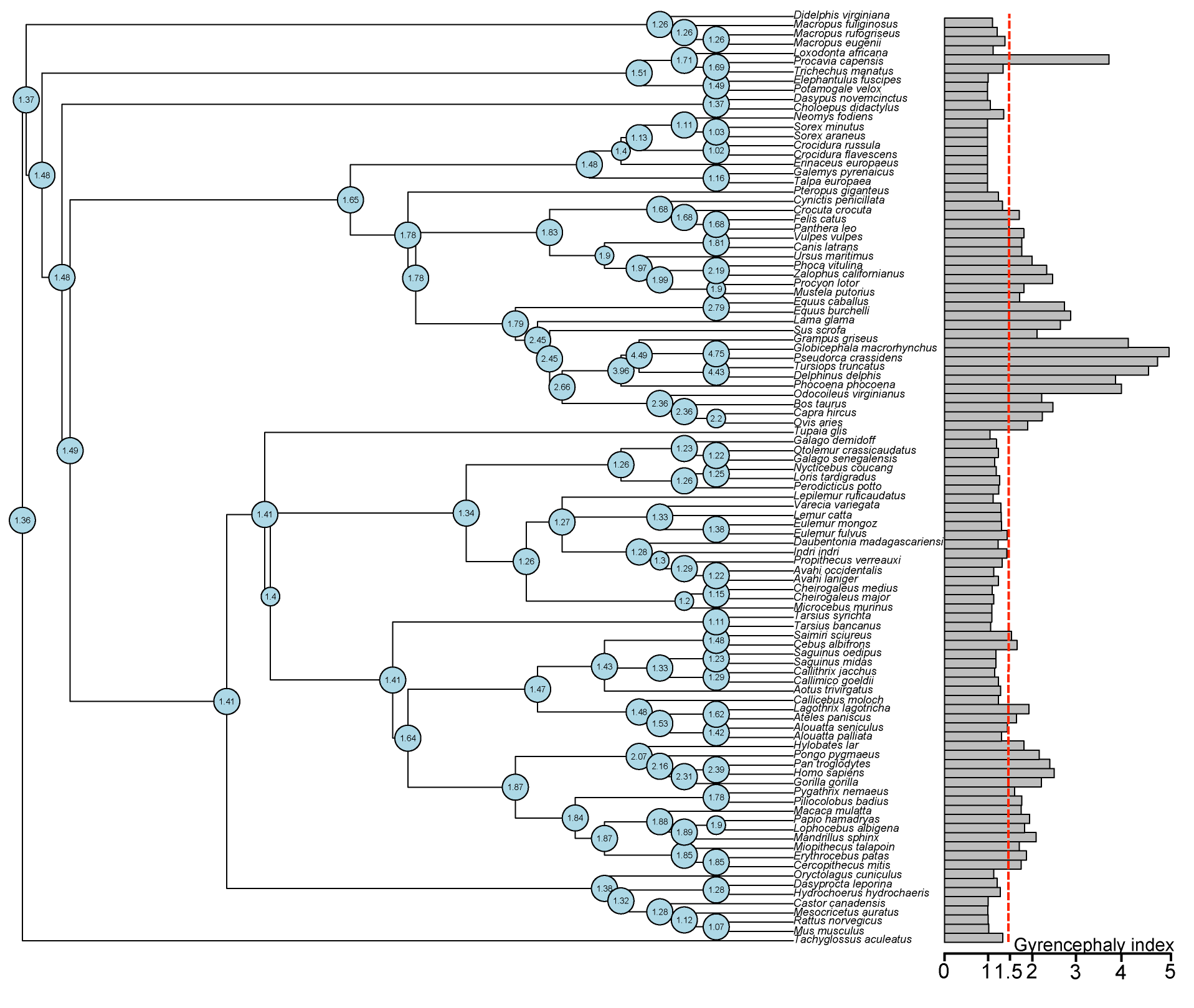

Supplement: Figure S2 — Maximum-likelihood ancestral node reconstruction of GI values at all internal nodes of the mammalian phylogeny. Reconstruction based on a delta (δ = 2.635) selection model. Barplot shows the distribution of GI values across the phylogeny; dashed red line indicates GI = 1.5. See Table S1, column F, for GI values. (TIF) [file pbio.1002000.s002.tif]

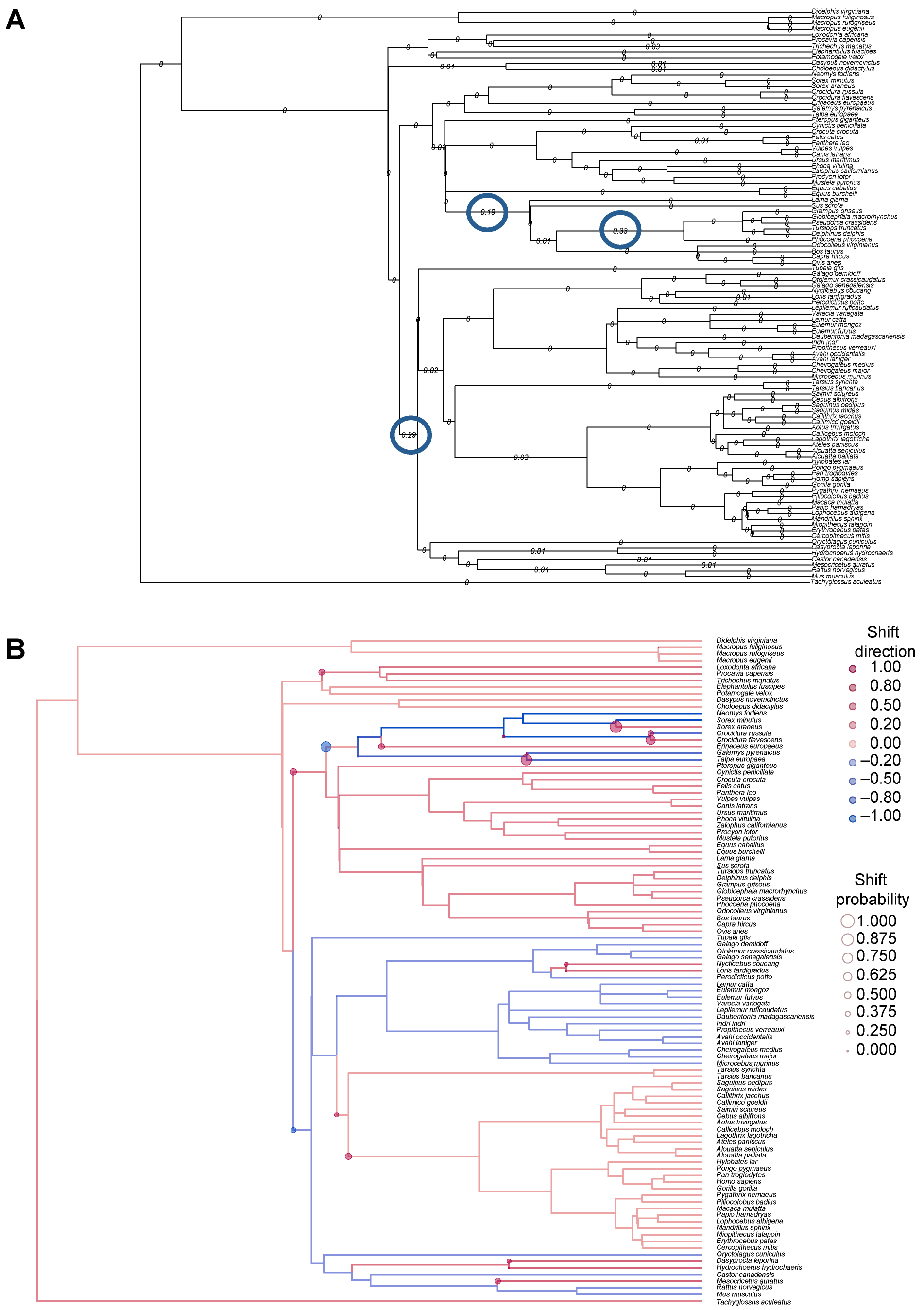

Supplement: Figure S3 — Rate-transitions in the mutation rate of GI values along lineages of the mammalian phylogeny. (A) A two-mode selection model that weights low over high root-to-tip substitutions. Numbers on the branches indicate the change in mutation-rate compared to the previous branch: 0 values indicate no significant change; values >0 indicate significant change (p<0.05). Note the especially high rate-transitions leading to primates, cetartiodactyls, and cetaceans (open blue circles). (B) Mutation- and transition-rate estimates of GI values using an Ornstein-Uhlenbeck selection model. Branches are colored to illustrate whether the mutation-rate estimates along each lineage are above (red) or below (blue) the median rate (orange); nodes are circled to indicate the posterior support of a transition-rate-shift event. The gradient of colors (see key) indicates the degree of deviation of the mutation-rate estimates (branches) and transition-rate estimates (nodes) from the median, with the highest deviation being arbitrarily set to ±1.0 and the median to 0.0; the size of the circles (see key) at the nodes indicates the degree of posterior support for a transition-rate-shift event, with the highest value being arbitrarily set to 1.0 and lack of support to 0.0. Note that simians have evolved GI values at a rate consistent with the mammalian median. See Table S1, column F, for GI values. (TIF) [file pbio.1002000.s003.tif]

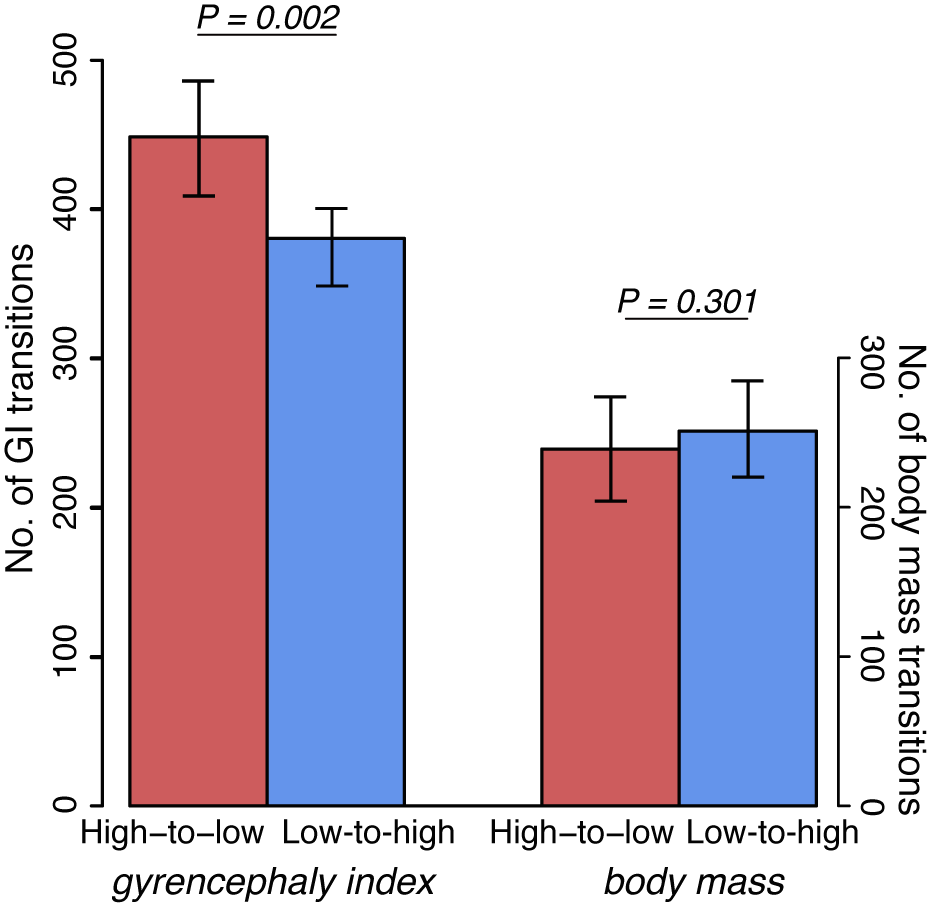

Supplement: Figure S4 — Barplots of types of transitions over mammalian evolution between four GI groups (see Figure 2A ) and between five body-mass groups (see Tables S1 and S2) averaged over 105 simulations. The number of total transitions from one GI (left) or body mass (right) group to another is summed as either high-to-low or low-to-high transitions. Note that significantly more high-to-low than low-to-high transitions are observed for GI, but that no significant difference in type of transition is observed for body mass. Error bars, standard error of the mean (SEM). (TIF) [file pbio.1002000.s004.tif]

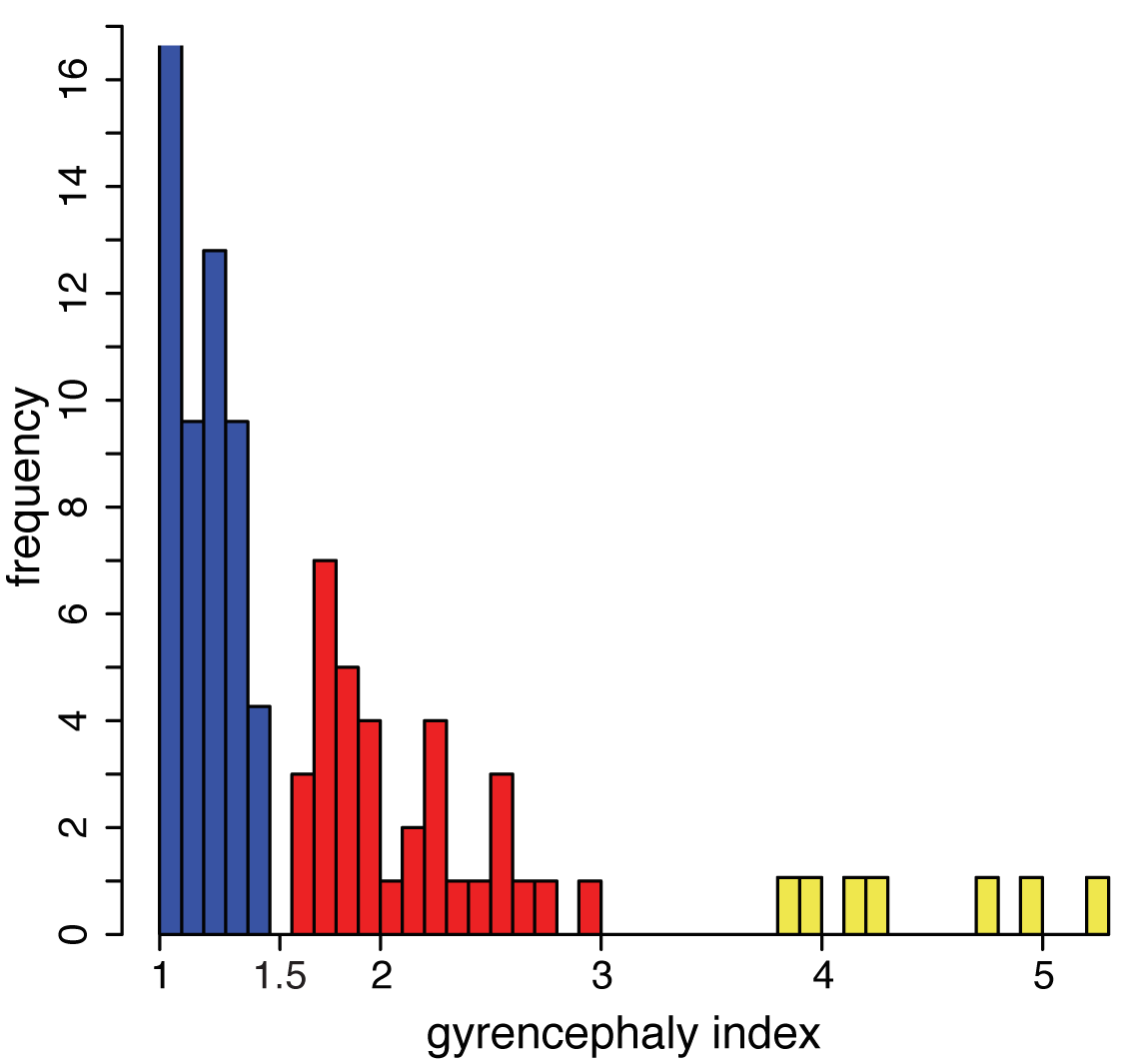

Supplement: Figure S5 — The bimodal distribution of GI values across the mammalian phylogeny is non-random. A histogram showing the frequency of occurrence of GI values, binned at 0.1 intervals, for the 102 mammalian species listed in Table S1. Blue, GI values ≤1.5; red, GI values >1.5. The bimodal distribution of GI values shows a natural break at GI = 1.5, which is supported by energy-based hierarchical clustering (see Figure 2B). Note the possibility for a third GI group (GI>3, yellow), constituting cetaceans and elephant; however, we have too few sampled species from these orders to assess the group decisively (see Figure S10). See Table S1, column F, for GI values. (TIF) [file pbio.1002000.s005.tif]

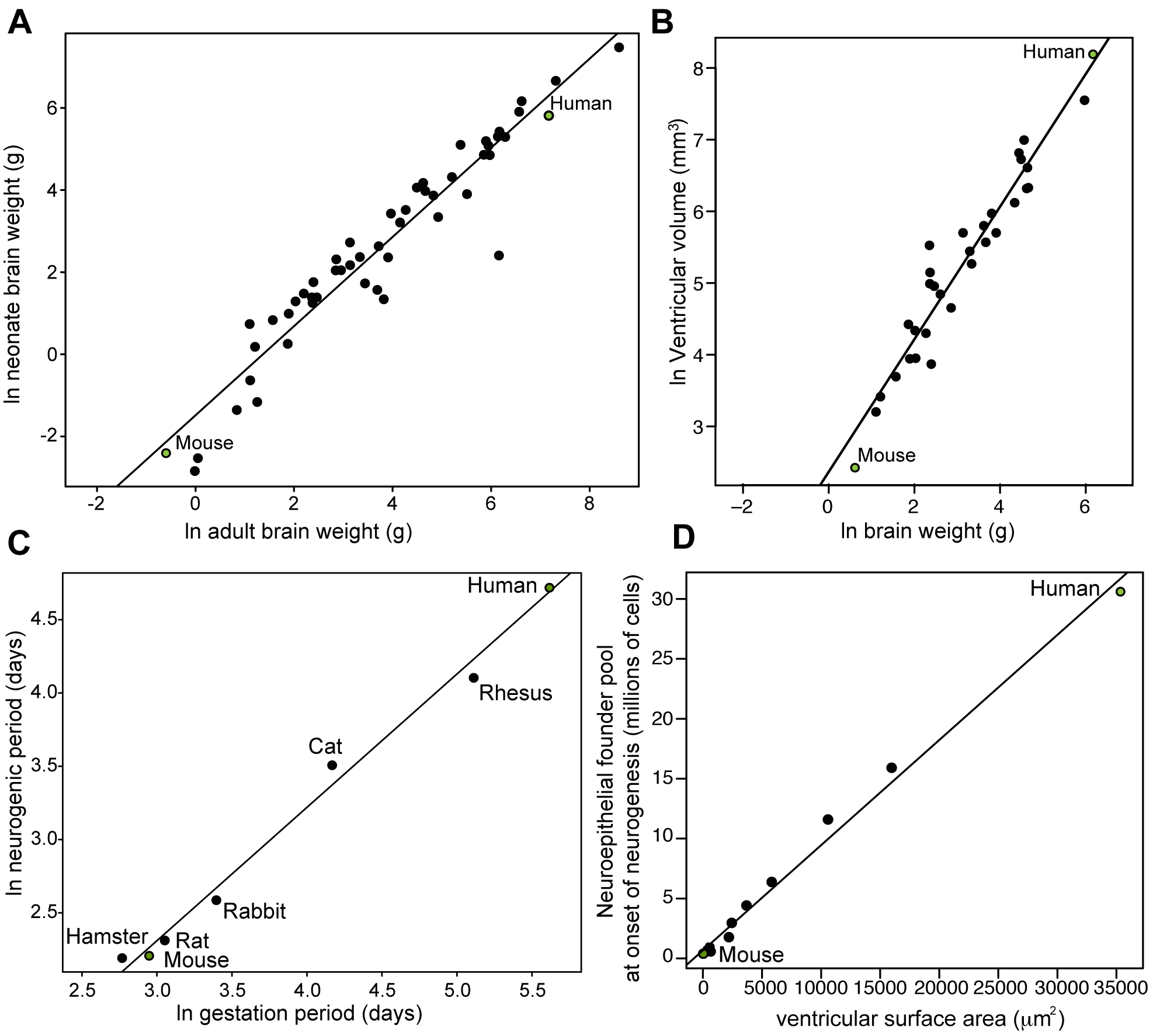

Supplement: Figure S6 — Ln-transformed plots of neonate brain weight (A) and ventricular volume (B) as functions of adult brain weight; neurogenic period as a function of gestation period (C); and a plot of neuroepithelial founder cells as a function of ventricular surface area (C). (A) Neonate brain weight scales linearly with adult brain weight for 52 eutherian species (y = 1.09x−1.49, R2 = 0.92, p = 6×10^−7^). (B) Ventricular volume scales linearly with adult brain weight for 30 eutherian species (y = 0.93x+2.37, R2 = 0.93, p = 9×10^−8^). (C) Neurogenic period scales linearly with gestation period for a sample of seven species (y = 0.91x−0.42, R2 = 0.94, p = 0.0002), spanning two mammalian superorders. (D) Ventricular surface area, converted from ventricular volume (see Estimating neuroepithelial founder pool populations), scales linearly with our estimated neuroepithelial founder populations (y = 6.7×10^5^+878x, R2 = 0.94, p = 5×10^−8^). (A, C) Note that these plots demonstrate the strong predictive powers of adult brain weight and gestation period for neonate brain weight and neurogenic period, respectively, validating the assumptions made in Figure 4. Mouse and human are indicated by filled green circles. See Table S1 for primary data. (TIF) [file pbio.1002000.s006.tif]

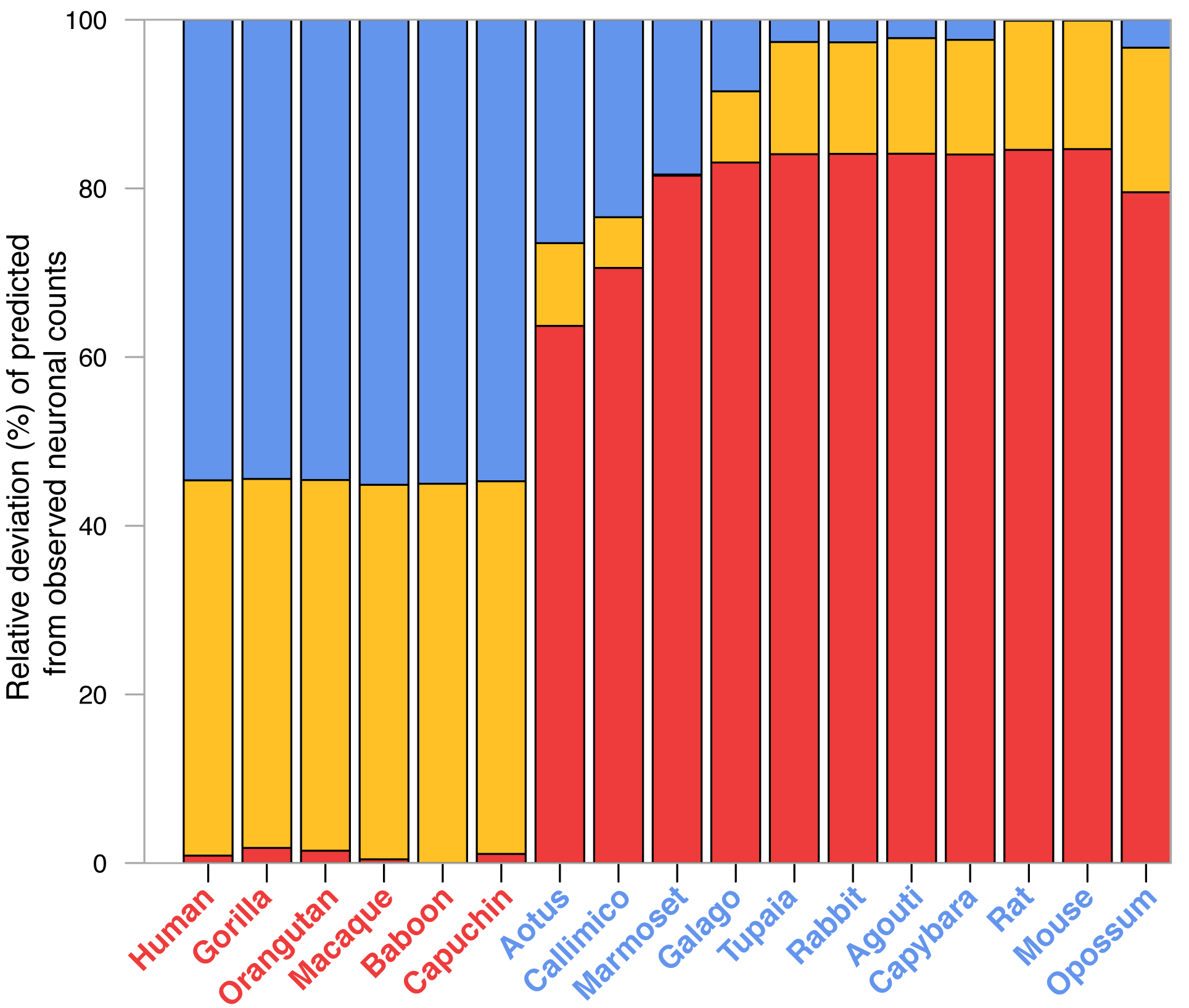

Supplement: Figure S7 — Stacked barplot, for the indicated species, of deviations between the observed cortical neuron counts and those predicted based on human (red), mouse (blue), and marmoset (yellow) neurogenic programs (see Figure 5 and Table 2 ). For each species, deviations were calculated as |100 * ((Predicted−Observed)/Observed)| and then divided by the sum of deviations obtained for all three programs. Predictions based on the marmoset program deviate from observed neuron counts considerably for the six species with a GI value >1.5 (red text), but also slightly for species with a GI≤1.5 (blue text), indicating a necessity for differential proportional occurrences of bRG in low-GI species. It is worth noting that natural intraspecific variation in cortical neuron number has been shown to be considerably less than interspecific variation [89],[90]. See Table S8 for primary data. (TIF) [file pbio.1002000.s007.tif]

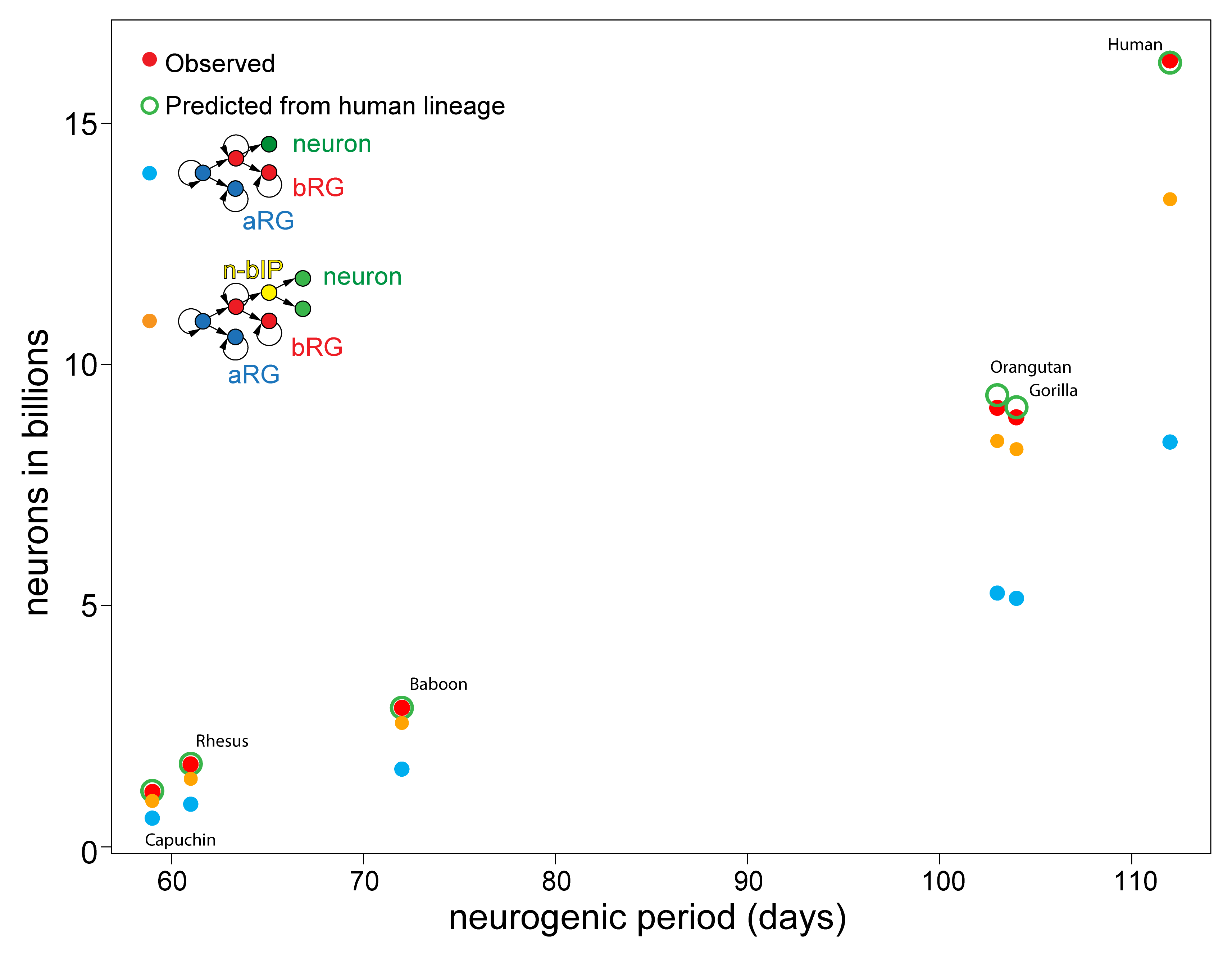

Supplement: Figure S8 — Plot of observed cortical neuron number (red circles) as a function of neurogenic period for six species with a GI value >1.5. Predicted neuron numbers are presented for the human neurogenic program (green circles; see Figure 5, Table 2) and for two further lineages, each of which is assumed to have a 100% proportional occurrence: direct neurogenesis from bRG (blue circles) and indirect neurogenesis from bRG via a self-consuming bIP cell (orange circles). Note that indirect neurogenesis from bRG via n-bIP is nearly sufficient to achieve the observed neuron count in the Capuchin monkey, but not that of human. See Tables S1 and S8 for primary data. (TIF) [file pbio.1002000.s008.tif]

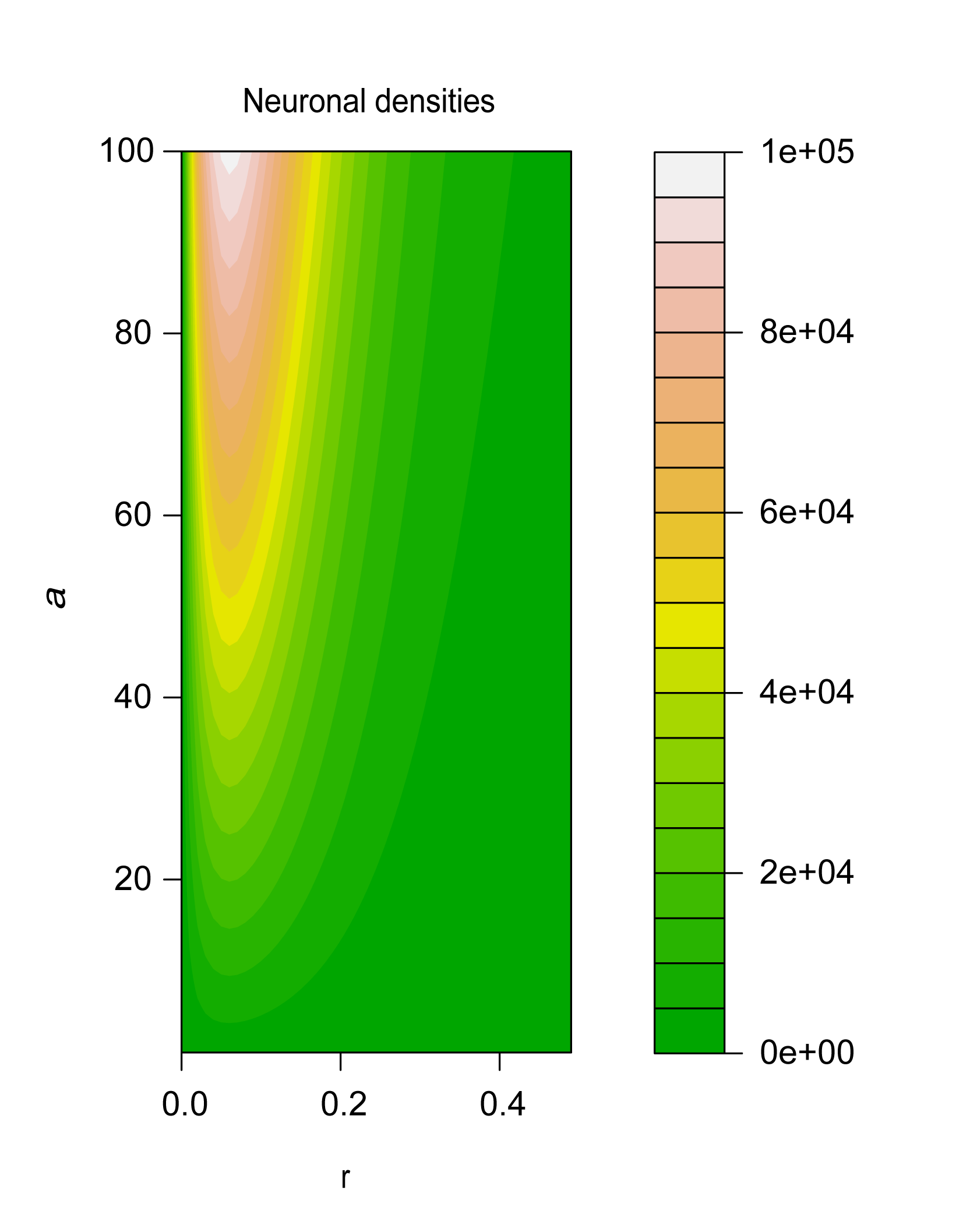

Supplement: Figure S9 — Neuron outputs from solutions to ODEs describing direct versus indirect neurogenesis for growth-rate values ≤0.5. Contour plot of neuron densities, b(t), for a varying initial asymmetrically dividing cell population, a, and likelihood of direct (r = 1) versus indirect (r = 0) neurogenesis. Note that neuron output increases maximally when both the initial cell pool increases (a→100) and the likelihood of indirect neurogenesis increases (r→0). Here, b(0) = 2, c(0) = 2, and t = 10. The optimal r to maximize b(t) approaches 0.5t when t is fixed. (TIF) [file pbio.1002000.s009.tif]

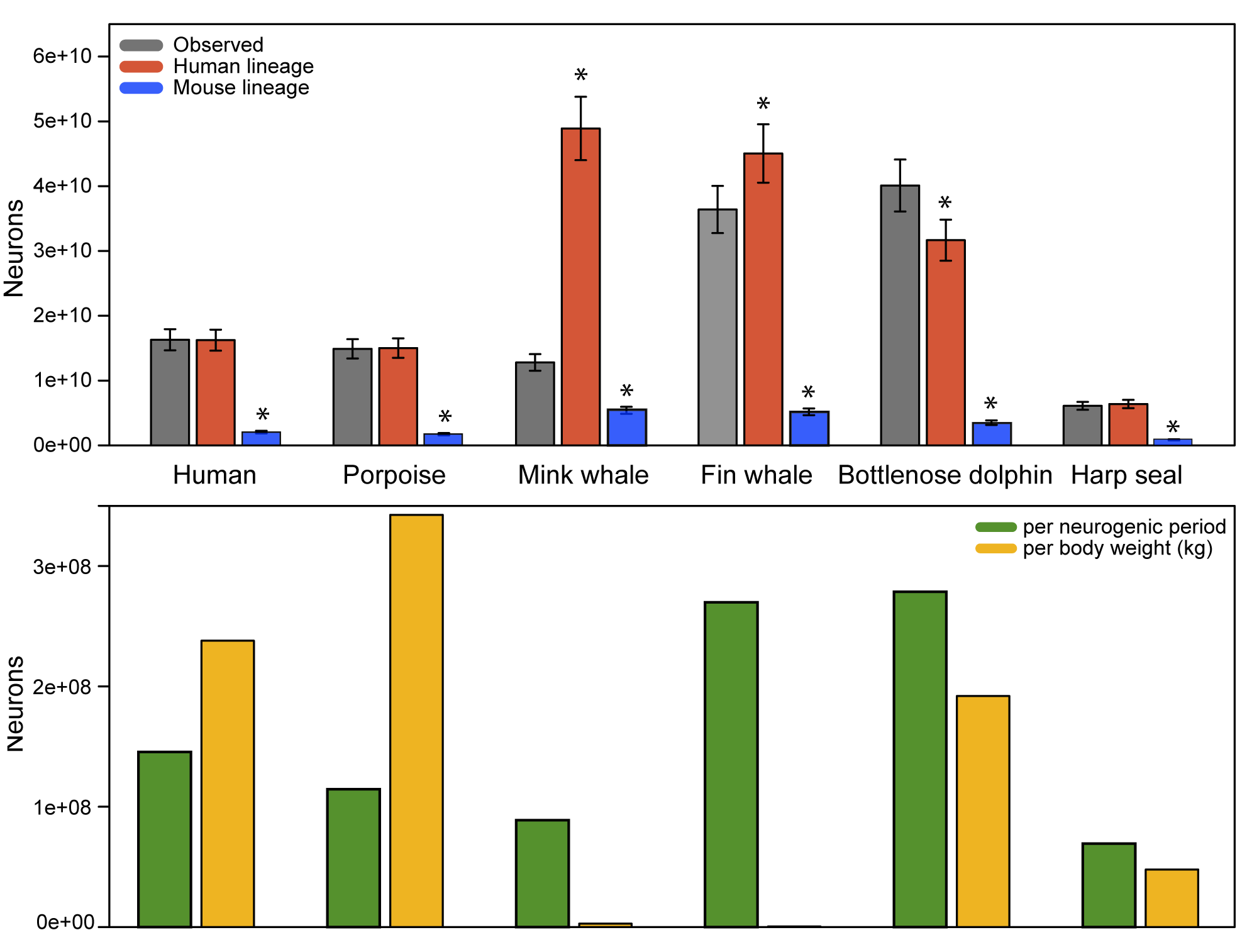

Supplement: Figure S10 — Neocortical development in marine mammals may be largely explained by the same neurogenic program as terrestrial mammals. (Top) Observed cortical neuron numbers for human, four cetacean species, and one marine carnivore (Harp seal, Pagophilus groenlandicus) are shown beside neuron numbers calculated from the human (red) and mouse (blue) neurogenic programs. Asterisks denote neuron numbers that are significantly different (T>7, p<0.05) from the observed; error bars are 95% confidence intervals. Note that the Bottlenose dolphin (Tursiops truncatus) is the only species for which the human program is not sufficient to achieve its observed number of neurons. (Bottom) The number of neurons generated per neurogenic day (green) and per body weight (gold) in human and the five marine mammals. Although the fin whale generates more neurons per neurogenic day, the human program produces a higher neuron count due to the fin whale's large estimated founder pool. See Tables S1 and S7 for primary data. (TIF) [file pbio.1002000.s010.tif]

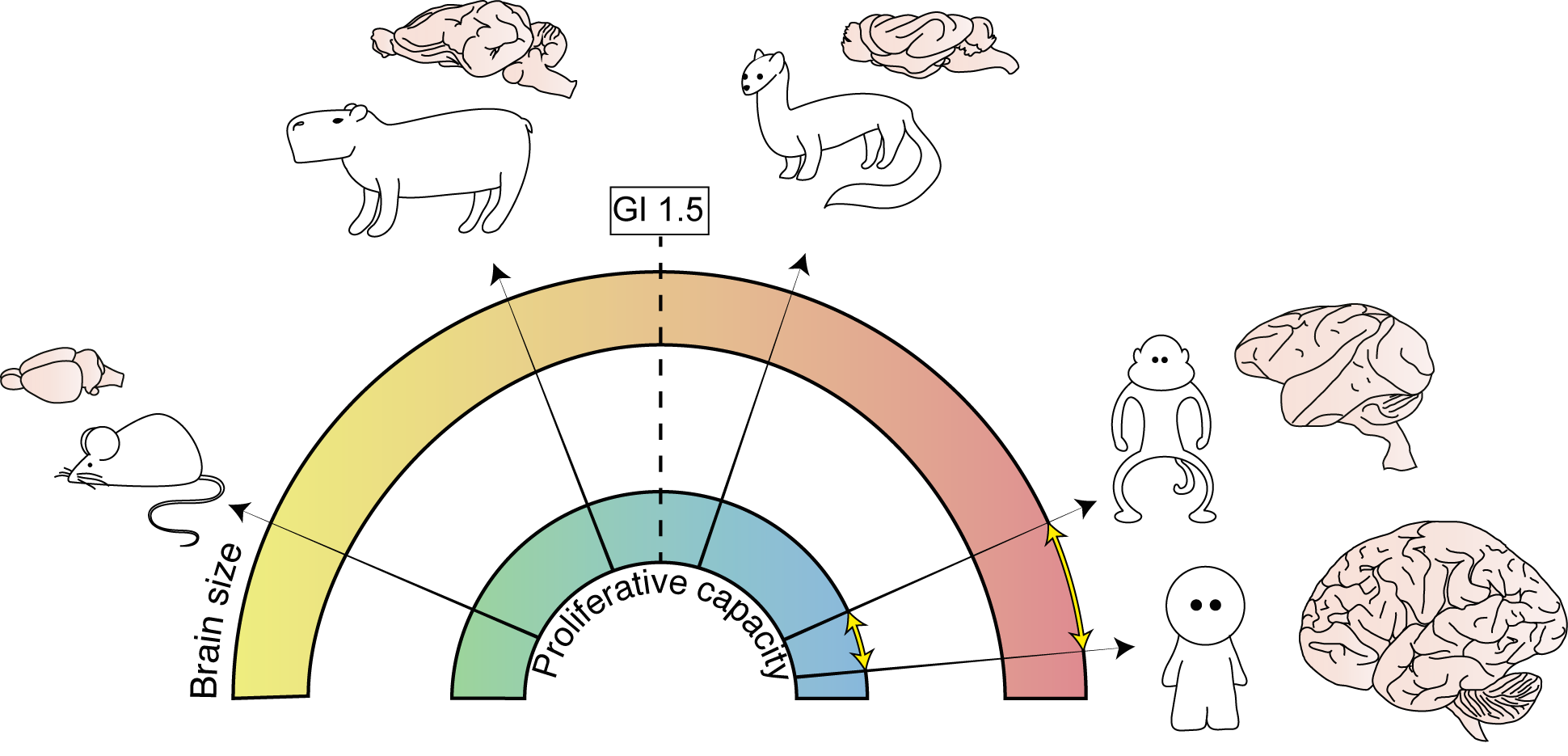

Supplement: Figure S11 — Neocortical complexity, represented here as cortical gyrification, is tightly linked to progenitor behavior in the SVZ. The nature of the link is such that incremental changes to SVZ progenitor behavior (inner ring) may effect exponential changes in neocortical complexity (outer ring). Therefore, only minor changes in the proliferative capacity of BPs (yellow arrow, inner ring) are needed to distinguish the major differences in neocortical complexity (yellow arrow, outer ring) between the macaque and human. It remains to be shown whether shifts in the proliferative capacity of BPs and neocortical complexity can occur independently (i.e., whether the black arrows can be bent). Pictured clockwise: mouse, capybara, ferret, macaque, human. (TIF) [file pbio.1002000.s011.tif]

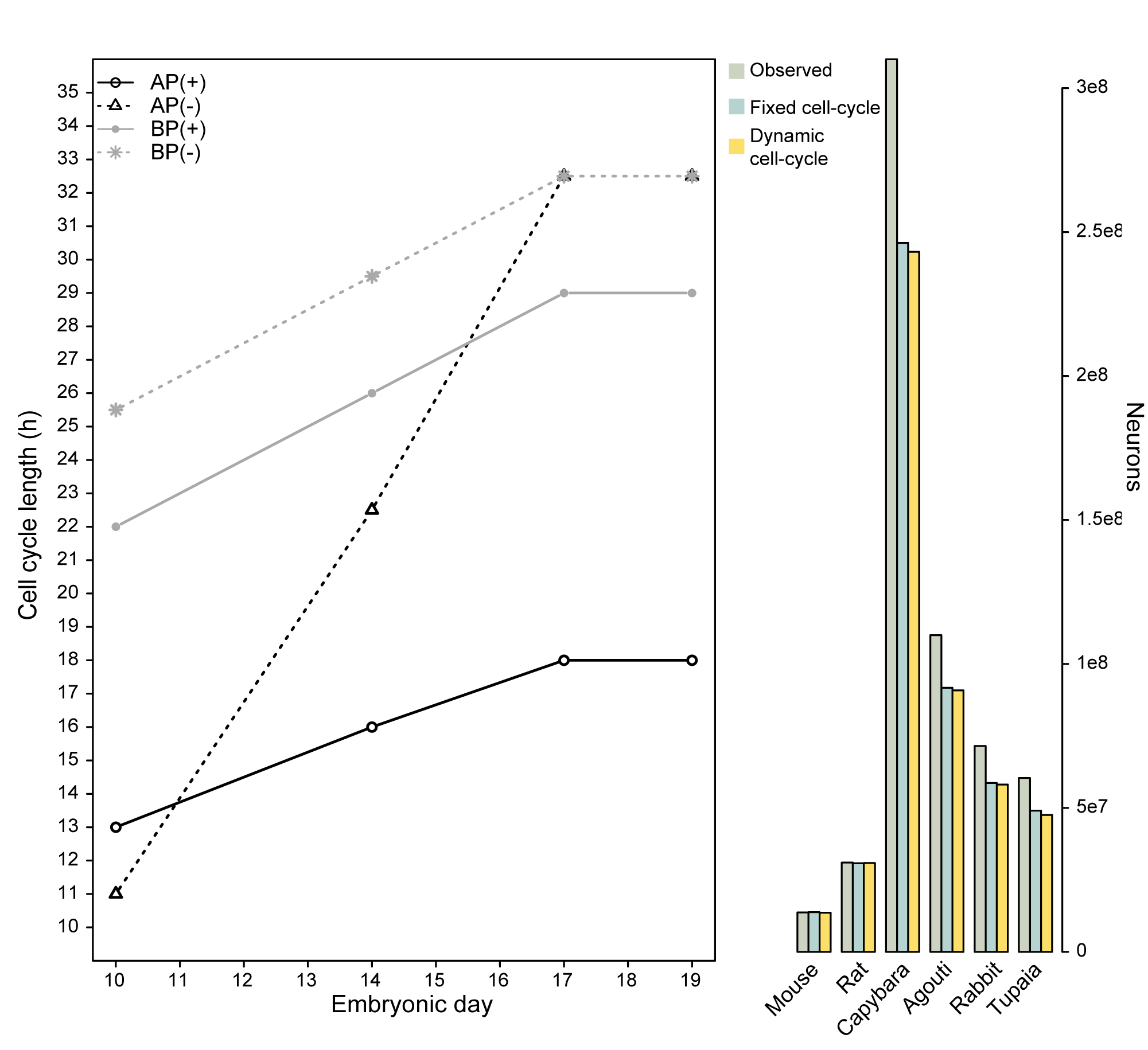

Supplement: Figure S12 — Cell-cycle length of cortical progenitors and neuron number predictions in non-primates. (A) Cell-cycle length of mouse Tis21-GFP–positive (+) and –negative (−) apical (AP) and basal (BP) progenitors at different stages of neurogenesis. Values for AP(−) and AP(+) at embryonic day (E) 10.5 (onset of mouse cortical neurogenesis) are taken from [79], values for AP(−), AP(+), BP(−), and BP(+) at E14.5 (mid-neurogenesis) are from [80], and the other values are extrapolated considering the data of [78]. (B) Barplot of the observed number of neurons (grey columns) in the cortex of four rodents, the rabbit, and Tupaia (a sister species to primates) compared to the number of neurons predicted using a fixed cell-cycle length value of 18.5 hours (blue columns, Table 1, i.e., the averaged cell-cycle length for apical and BPs from the mouse), as was done in Figure 5C, and the number of neurons predicted using dynamic cell-cycle length values for each progenitor class as shown in (A) (yellow columns). Note that for all species the predictions based on fixed and dynamic cell-cycle length values deviate from each other by <1%. The percentage deviations between observed and mouse neurogenic program-predicted neuron numbers are listed in Table S4. (TIF) [file pbio.1002000.s012.tif]
