# Supplementary material for: An Adaptive Threshold in Mammalian Neocortical Evolution
Source: PLoS Biol. 2014 Nov 18;12(11):e1002000. doi: 10.1371/journal.pbio.1002000 (PMC4236020; doi:10.1371/journal.pbio.1002000)
Supplement: Database S1 — The GI and life-history trait database (102 species) was assembled from the literature (see references 1–15 in Database S1). (DOC) [file pbio.1002000.s021.doc]

**External Database S1**

The GI and life history trait dataset (102 species in total) was assembled from the following sources: (1-15), AnAge database (<http://genomics.senescence.info/species/>), and PANTHERIA database (16). Additional data was collected from the references listed in the table (see below).

| Species | Data | Reference(s) |
| --- | --- | --- |
| *Alouatta paliatta* | Adult body (7670 g) and brain (54 g) weight, neonate body (440 g) and brain (30.800 g) weight  Neocortex volume (- corpus callosum) | (11)  (17) |
| *Aotus trivirgatus* | Neonatal brain weight (10.1 g)  Neocortex volume (- corpus callosum)  Cortical thickness | (18)  (17)  (19) |
| *Avahi laniger* | Brain weight  Max home range (1-2 ha)  Weight at birth (29 g), weight at weaning (500 g) | (20)  (21)  (22) |
| *Avahi occidentalis* | Activity timing (N), group size (<9), home range (1-2 ha) | (21) |
| *Bos taurus* | Adult body and brain weight, neonate body  and brain weight  Neonatal brain size (203 g), neonatal body mass (28366.67 g), maternal body mass (483502 g), gestation length (282 days), precocial  Medulla, cerebellum, mesencephalon, diencephalon, striatum, septum, amygdala, paleocortex, hippocampus, schizocortex, isocortex volume  Cortical thickness | (11)  (23)  (24)  (19) |
| *Callimico goeldii* | Neonate brain weight (5.8 g) | (25) |
| *Callithrix jacchus* | Cortical thickness  Neurogenic period (58 days) | (19)  (Kelava, personal observation) |
| *Canis latrans* | Adult body (8510 g) and brain (84.24 g) weight  Neocortex volume (- corpus callosum)  Medulla, cerebellum, mesencephalon, diencephalon, striatum, septum, amygdala, paleocortex, hippocampus, schizocortex, isocortex volume | (26)  (17)  (24) |
| *Capra hircus domestica* | Adult body and brain weights, neonate  body and brain weights  Neonatal brain size (45.53 g), neonatal body mass (1307.6 g), maternal body mass (23333.333 g), litter size (1.25), gestation length (154.748 days), precocial | (11)  (23) |
| *Castor canadensis* | Adult body (20000 g) and brain (40 g) weight, neonate body (472 g) and brain (13.900 g) weight | (11) |
| *Cheirogaleus major* | Mating system (monogamous), body weight (414 g), max home range (4.4 ha) | (27) |
| *Cheirogaleus medius* | Resting metabolic rate | (28) |
| *Choloeps didactylus* | Brain weight (7.7 g)  Neocortex volume (- corpus callosum)  Medulla, cerebellum, mesencephalon, diencephalon, striatum, septum, amygdala, paleocortex, hippocampus, schizocortex, isocortex volume | (29)  (17)  (24) |
| *Crocidura flavescens* | Brain weight (0.41 g)  Cortical thickness | (30)  (19) |
| *Crocidura russula* | Social system (monogamous)  Cortical thickness  Brain weight (0.019 g) | (31)  (19)  (12) |
| *Crocuta crocuta* | Neocortex volume (- corpus callosum)  Medulla, cerebellum, mesencephalon, diencephalon, striatum, septum, amygdala, paleocortex, hippocampus, schizocortex, isocortex volume | (17)  (24) |
| *Cynictis penicillata* | Body weight (589 g), weaning (40 d), altricial, litter size (1.8), group size (3.6=<9), mating system (polygynandry), above ground dwelling, trophic level (carnivore)  Litters per year (2) | (32)  (33) |
| *Dasyprocta leporine* | Social system (monogamous) | (34) |
| *Dasypus novemcinctus* | Adult body (3700 g) and brain (12 g) weight, neonate body (80 g) and brain weight (3.500 g)  Adult body and brain weight  Medulla, cerebellum, mesencephalon, diencephalon, striatum, septum, amygdala, paleocortex, hippocampus, schizocortex, isocortex volume | (11)  (26)  (24) |
| *Daubentonia madagascariensis* | Resting metabolic rate (1393.9 mL O_2_/h) | (28) |
| *Delphinus delphis* | Brain weight  Trophic level (carnivore)  Neonatal brain weight (476 g) | (35)  (36)  (37) |
| *Didelphis virginiana* | Brain weight (6.72 g) | (38) |
| *Elephantulus fuscipes* | Brain weight (1.33 g), body weight (57 g) | (12) |
| *Equus burchelli* | Medulla, cerebellum, mesencephalon, diencephalon, striatum, septum, amygdala, paleocortex, hippocampus, schizocortex, isocortex volume | (24) |
| *Equus caballus* | Adult body and brain weight, neonate body  and brain weight  Neocortex volume (- corpus callosum)  Cortical thickness | (11)  (17)  (19) |
| *Erinaceus europaeus* | Adult body (928 g) and brain (3.50 g) weight, neonate body (13.90 g) and brain (0.313 g) weight  Altricial, group size (1) | (11)  (39) |
| *Eulemur mongoz* | Diet (frugivorous), % fruit and seeds  Group size (<9, pair+offspring), acivity timing (N + D, depending on the season and area), weight at birth (60-70 g for the genus Eulemur)  Resting metabolic rate  Neocortex volume (- corpus callosum) | (40)  (41)  (28)  (17) |
| *Felis catus* | Brain weight, gestation length  Adult body (2500 g) and brain weight (28.40 g), neonate body (114 g) and brain weight (5.600 g)  Neonatal brain size (5.65 g), neonatal body mass (105.25 g), maternal body mass (3150 g), gestation length (64 days), altricial  Neurogenic period (33.5 days)  Cortical thickness | (42)  (11)  (23)  (43)  (19) |
| *Galago demidovii* | Adult brain size (2.7 g)  Resting metabolic rate  Cortical thickness | (44)  (28)  (19) |
| *Galago senegalensis* | Neocortex volume (- corpus callosum)  Social system (monogamous)  Cortical thickness | (17)  (45)  (19) |
| *Galemys pyrenaicus* | Brain weight (1.33 g) | (12) |
| *Globicephala macrorhynchus* | Brain weight (2886.296 g) (brain volume*1.036)  Cortical thickness | (46)  (19) |
| *Gorilla gorilla* | Adult body (140000 g) and brain (406 g) weight, neonate body (1750 g) and brain (227 g) weight | (11) |
| *Grampus griseus* | Brain weight (1588 g)  Cortical thickness | (46)  (19) |
| *Hapalemur griseus* | Group size, activity (diurnal), mating system (polygyny)  Group size (3), activity, diet (folivorous)  Resting metabolic rate | (47)  (48)  (28) |
| *Homo sapiens* | Neurogenic period (112 days)  Cortical thickness  Corpus callosum | See text.  (19)  (30) |
| *Hydrochaeris hydrochaeris* | Adult body (29500 g) and brain (76 g) weight, neonate body (1200 g) and brain (24.800 g) weight  Social system (polygynous to promiscuous – harem; dominant males) | (11)  (49) |
| *Hylobates lar* | Adult body (5500 g) and brain (102 g) weight, neonate body (400 g) and brain (65 g) weight  Neocortex volume (- corpus callosum)  Corpus callosum | (11)  (17)  (30) |
| *Indri indri* | Sexual maturity (48-84 months, mean 2008 days)  Body weight (10500 g) | (50)  (44) |
| *Lama glama* | Adult body and brain weight, neonate body  and brain weight  Neonatal brain size (164.45 g), neonatal body mass (12000 g), maternal body mass (103000 g), gestation length (334.1 days), precocial  Medulla, cerebellum, mesencephalon, diencephalon, striatum, septum, amygdala, paleocortex, hippocampus, schizocortex, isocortex volume | (11)  (23)  (24) |
| *Lemur catta* | Body weight, group size (female dominated 6-24 individuals, average 11-16)  Body weight (2233 g), litter size (1.3), weaning period (135 days), inter-litter interval (511 days), sexual maturity (550 days)  Adult body (2100 g) and brain (22 g) weight, neonate body (78 g) and brain (8.780 g) weight  Resting metabolic rate  Weight at weaning (1080 g)  Neocortex volume (- corpus callosum) | (51)  (44)  (11)  (28)  (22)  (17) |
| *Lemur fulvus* | Sexual maturity (548 days), maximum longevity (36 years)  Weight at weaning (1350 g) | (41)  (22) |
| *Lepilemur mustelinus* | Home range (1-3 ha), female and male mass | (52) |
| *Lepilemur ruficaudatus* | Gestation length (176 days), body weight, activity, diet, group size, litter size (1), home range (1 ha), inter-litter interval (365 days)  Weight at birth (27 g), weight at weaning (250 g) | (53)  (22) |
| *Loris tardigradus* | Resting metabolic rate | (28) |
| *Loxodonta africana* | Brain weights (4000-9000)  Adult body (2750000 g) and brain (4480 g) weight, neonate body (120000 g) and brain (1650 g) weight  Neocortex volume (- corpus callosum)  Cortical thickness  Carpus callosum | (54)  (11)  (55)  (19)  (30) |
| *Macaca mulatta* | Adult body (5980 g) and brain (84.60 g) weight, neonate body (540 g) and brain (58 g) weight  Neurogenic period (60.5 days)  Neocortex volume (- corpus callosum)  Cortical thickness | (11)  (56)  (17)  (19) |
| *Macropus eugenii* | Brain weight (23.7 g) | (38) |
| *Macropus fuliginosus* | Body length  Brain weight (64.80 g), body weight (31000 g) | (57)  (58) |
| *Macropus rufogriseus* | Brain weight (37.80 g), body weight (15200 g) | (58) |
| *Mandrillus sphinx* | Neocortex volume (- corpus callosum) | (17) |
| *Mesocricetus auratus* | Adult body (125 g) and brain (1.12 g) weight, neonate body (1.80 g) and brain (0.060 g) weight  Social system (multi-male), age at eye opening (13 d)  Neurogenic period (9 days) | (11)  (59)  (43) |
| *Monodelphis virgiana* | Brain (0.12 g) and neocortex (0.01 g) weight  Neurogenic period (10 d) | (60), (61) |
| *Microcebus murinus* | Body weight (84 g)  Neocortex volume (- corpus callosum) | (44)  (17) |
| *Mus musculus* | Adult body (24 g) and brain (0.45 g) weight, neonate body (1.50 g) and brain (0.090 g) weight  Body length, age at eye opening (14 d)  Neurogenic period (9 days) | (11)  (62)  (63), (64) |
| *Mustela putorius* | Neocortex volume (- corpus callosum) | (17) |
| *Neomys fodiens* | Brain weight (0.32 g)  Cortical thickness | (12)  (19) |
| *Nycticebus coucang* | Adult body (1230 g) and brain (12.80 g) weight, neonate body (30.50 g) and brain (4 g) weight  Resting metabolic rate  Neocortex volume (- corpus callosum) | (11)  (28)  (17) |
| *Odocoileus virginianus* | Neocortex volume (- corpus callosum)  Medulla, cerebellum, mesencephalon, diencephalon, striatum, septum, amygdala, paleocortex, hippocampus, schizocortex, isocortex volume | (17)  (24) |
| *Oryctolagus cuniculus* | Brain weight (3.4 g)  Adult body (2200 g) and brain weight (9.60 g), neonate body (38 g) and brain weight (1.290 g)  Social system (polygynandrous = multi-male)  Neurogenic period (13 days) | (65)  (11)  (66)  (43); Kelava and Huttner, in preparation) |
| *Otolemur crassicaudatus* | Adult body (700 g) and brain weight (9.90 g), neonate body (40 g) and brain weight (4 g)  Resting metabolic rate  Neocortex volume (- corpus callosum) | (11)  (28)  (17) |
| *Ovis aries* | Adult body and brain weight, neonate body  and brain weight  Neonatal brain size (43.65 g), neonatal body mass (2412.5 g), maternal body mass (33100 g), gestation length (151.31 days, precocial  Neocortex, striatum, corpus geniculatum laterale volume  Cortical thickness | (11)  (23)  (67)  (19) |
| *Pan troglodytes* | Adult body (45000 g) and brain (360 g) weight, neonate body (1560 g) and brain (128 g) weight  Neocortex volume (- corpus callosum) | (11)  (17) |
| *Panthera leo* | Adult body (1750000 g) and brain (260 g) weight, neonate body (1300 g) and brain (49.500 g) weight  Neonatal brain size (49.5 g), neonatal body mass (1383.9 g), maternal body mass (135500 g), litter size (2), altrici  Medulla, cerebellum, mesencephalon, diencephalon, striatum, septum, amygdala, paleocortex, hippocampus, schizocortex, isocortex volume | (11)  (23)  (24) |
| *Papio hamadryas* | Neocortex volume (- corpus callosum) | (17) |
| *Perodicticus poto* | Resting metabolic rate  Neocortex volume (- corpus callosum) | (28)  (17) |
| *Phoca vitulina* | Brain weight (273.75 g)  Medulla, cerebellum, mesencephalon, diencephalon, striatum, septum, amygdala, paleocortex, hippocampus, schizocortex, isocortex volume | (68)  (24) |
| *Phocoena phocoena* | Adult body (55500 g) and brain (537 g) weight, neonate body (6750 g) and brain (199 g) weight  Weight at weaning (30000 g)  Cortical thickness | (11)  (69)  (19) |
| *Pongo pygmaeus* | Adult body (36900 g) and brain (343 g) weight, neonate body (1500 g) and brain (129 g) weight | (11) |
| *Potamogale velox* | Brain weight (4.160 g) | (12) |
| *Procavia capensis* | Body weight (4300 g), group size (80), activity timing (diurnal), weight at birth (202 g)  Body weight (1800 g, 3600 g), brain weight (19 g, 18 g)  Adult body (3800 g) and brain weight (20.50g), neonate body (200 g) and brain weight (7.750 g)  Social system (harem)  Neocortex volume (- corpus callosum) | (70)  (29)  (11)  (71)  (17) |
| *Procyon lotor* | Adult body (5175 g) and brain (40 g) weight  Neonatal brain size (4.8 g), neonatal body mass (97.5 g), maternal body mass (6320 g), gestation length (64.025 days), litter size (2.89), altricial  Neocortex volume | (26)  (23)  (72) |
| *Propithecus verreauxi* | Resting metabolic rate  Weight at weaning (1000 g) | (28)  (22) |
| *Propithecus verreauxi* | Body size, group size (2-14), folivorous | (51) |
| *Pseudorca crassidens* | Brain weight (4444.50 g) | (73) |
| *Pteropus giganteus* | Social system (polygynandrous = multi-male), body length (230 mm) | (74) |
| *Pteropus giganteus* | Brain weight (9.0 g)  Body weight (875 g, 1084 g)  Body weight (1021 g), group size (900) | (75)  (76)  (77) |
| *Rattus norvegicus* | Adult body (339 g) and brain (2.38 g) weight, neonate body (4.92 g) and brain (0.280 g) weight  Body length (399 mm), social system (polygynandrous = multi male), activity timing (nocturnal and crepuscular), age at eye opening (15.5 days)  Neurogenic period (10 days)  Neocortex volume (- corpus callosum) | (11)  (78)  (43)  (17) |
| *Saimiri sciureus* | Neocortex volume (- corpus callosum)  Cortical thickness | (17)  (19) |
| *Sorex araneus* | Brain weight (0.20 g)  Cortical thickness | (12)  (19) |
| *Sorex minutus* | Brain weight (0.11 g)  Cortical thickness | (12)  (19) |
| *Sus scrofa domestica* | Adult body and brain weight, neonate body  and brain weight  Neonatal brain size (26.6 g), neonatal body mass (1030 g), maternal body mass (75200 g), gestation length (118.29 days), litter size (5.375, precocial  Brain parts volumes  Cortical thickness | (11)  (23)  (79)  (19) |
| *Tachyglossus aculeatus* | Brain weight (27.5 g), body weight (4720 g) |  |
| *Talpa europaea* | Mating system (solitary), age of eye opening (22 days)  Brain weight (1.02 g) | (80)  (12) |
| *Tarsius bancanus* | Weight at birth (28.5 g)  Lifespan (12 years) | (81)  (44) |
| *Tarsius syrichta* | Max home range (0.6-2 ha, mean 1.3 ha), group size (<9)  Neocortex volume (- corpus callosum) | (82)  (17) |
| *Trichechus manatus* | Body size, brain size, sexual maturity, litter size, interbirth interval, longevity, neonate brain mass, neonate body mass, precocial  Social system (multi-male)  Medulla, cerebellum, mesencephalon, diencephalon, striatum, septum, amygdala, paleocortex, hippocampus, schizocortex, isocortex volume  Cortical thickness | (83)  (84)  (24)  (83) |
| *Tupaia glis* | Age at first breeding (4.5 months), gestation length (40-52 days), weaning (25-35 days), home range (7255-10174 m2, taken the mean of 0.08715 km2)  Body weight (170 g), brain weight (3.2 g)  Brain weight (2.752 g)  Group size (1-solitary)  Adult body (150 g) and brain (3.15 g) weight, neonate body (9.20 g) and brain (0.530 g) weight  Social system (monogamous), altricial offspring, age of eye opening (10 days)  Cortical thickness | Wikipedia  (12)  (85)  (86)  (11)  (87)  (19) |
| *Tursiops truncates* | Brain weight (1378 g)  Adult body (155000 g) and brain (1600 g) weight, neonate body (20000 g) and brain (770 g) weight  Social system (polygamous=multi-male)  Cortical thickness | (37)  (46)  (88)  (19) |
| *Ursus maritimus* | Adult body (250000 g) and brain (500 g) weight, neonate body (590 g) and brain (11.100 g) weight  Neonatal brain size (11.09 g), neonatal body mass (668.01 g), maternal body mass (272500 g), litter size (1.33), altricial  Medulla, cerebellum, mesencephalon, diencephalon, striatum, septum, amygdala, paleocortex, hippocampus, schizocortex, isocortex volume | (11)  (23)  (24) |
| *Varecia variegate* | Resting metabolic rate  Weight at weaning (2500 g) | (28)  (22) |
| *Vulpes vulpes* | Adult body (6000 g) and brain (48 g) weight, neonate body (105 g) and brain (3.820 g) weight  Neonatal brain size (3.82 g), neonatal body mass (103.06267 g), maternal body mass (3900 g), litter size (4.875), altricial  Medulla, cerebellum, mesencephalon, diencephalon, striatum, septum, amygdala, paleocortex, hippocampus, schizocortex, isocortex volume  Cortical thickness | (11)  (23)  (24)  (19) |
| *Zalophus californianus* | Neocortex volume (- corpus callosum)  Medulla, cerebellum, mesencephalon, diencephalon, striatum, septum, amygdala, paleocortex, hippocampus, schizocortex, isocortex volume | (17)  (24) |

1. Zilles K, Armstrong E, Moser KH, Schleicher A, & Stephan H (1989) Gyrification in the cerebral cortex of primates. (Translated from eng) *Brain Behav Evol* 34(3):143-150 (in eng).

2. Walker R, Burger O, Wagner J, & Von Rueden CR (2006) Evolution of brain size and juvenile periods in primates. (Translated from eng) *Journal of human evolution* 51(5):480-489 (in eng).

3. Martin RD (2007) The evolution of human reproduction: a primatological perspective. (Translated from eng) *Am J Phys Anthropol* Suppl 45:59-84 (in eng).

4. Sawaguchi T (1988) Correlations of cerebral indices for 'extra' cortical parts and ecological variables in primates. (Translated from eng) *Brain Behav Evol* 32(3):129-140 (in eng).

5. Marino L (2007) Cetacean brains: how aquatic are they? (Translated from eng) *Anatomical record* 290(6):694-700 (in eng).

6. Hassiotis M, Paxinos G, & Ashwell KW (2003) The anatomy of the cerebral cortex of the echidna (Tachyglossus aculeatus). (Translated from eng) *Comparative biochemistry and physiology. Part A, Molecular & integrative physiology* 136(4):827-850 (in eng).

7. Neal J*, et al.* (2007) Insights into the gyrification of developing ferret brain by magnetic resonance imaging. (Translated from eng) *Journal of anatomy* 210(1):66-77 (in eng).

8. Harvey PH & Cluttonbrock TH (1985) Life-History Variation in Primates. (Translated from English) *Evolution* 39(3):559-581 (in English).

9. Gittleman JL (1986) Carnivore Brain Size, Behavioral Ecology, and Phylogeny. (Translated from English) *J Mammal* 67(1):23-36 (in English).

10. Pillay P & Manger PR (2007) Order-specific quantitative patterns of cortical gyrification. (Translated from eng) *Eur. J. Neurosci.* 25(9):2705-2712 (in eng).

11. Sacher GA & Staffeld EF (1974) Relation of Gestation Time to Brain Weight for Placental Mammals - Implications for Theory of Vertebrate Growth. (Translated from English) *Am. Nat.* 108(963):593-615 (in English).

12. Stephan H, Frahm H, & Baron G (1981) New and revised data on volumes of brain structures in insectivores and primates. (Translated from eng) *Folia Primatol (Basel)* 35(1):1-29 (in eng).

13. Lewitus E, Sherwood CC, & Hof PR (2012) Cellular signatures in the primary visual cortex of phylogeny and placentation. (Translated from eng) *Brain Struct Funct* 217(2):531-547 (in eng).

14. Shultz S & Dunbar RI (2007) The evolution of the social brain: anthropoid primates contrast with other vertebrates. (Translated from eng) *Proc Biol Sci* 274(1624):2429-2436 (in eng).

15. Kappeler PM & Pereira ME (2003) *Primate Life Histories and Socioecology* (University of Chicago Press).

16. Jones KE*, et al.* (2009) PanTHERIA: a species-level database of life history, ecology, and geography of extant and recently extinct mammals. *Ecology* 90(2648).

17. Bush EC & Allman JM (2003) The scaling of white matter to gray matter in cerebellum and neocortex. (Translated from eng) *Brain Behav. Evol.* 61(1):1-5 (in eng).

18. Parker ST & Gibson KR (1994) *'Language' and Intelligence in Monkeys and Apes: Comparative Developmental Perspectives* (Cambridge University Press).

19. Hofman MA (1985) Size and shape of the cerebral cortex in mammals. I. The cortical surface. *Brain Behav. Evol.* 27(1):28-40.

20. Karpanty SM (2006) Direct and indirect impacts of raptor predation on lemurs in southeastern Madagascar. (Translated from English) *Int J Primatol* 27(1):239-261 (in English).

21. Ganzhorn JrU, Abraham JP, & Razanahoera-Rakotomalala Mn (1985) Some aspects of the natural history and food selection of Avahi laniger. *Primates* 26(4):452-463.

22. Godfrey LR, Samonds KE, Jungers WL, Sutherland MR, & Irwin MT (2004) Ontogenetic correlates of diet in Malagasy lemurs. (Translated from eng) *Am. J. Phys. Anthropol.* 123(3):250-276 (in eng).

23. Capellini I, Venditti C, & Barton RA (2011) Placentation and maternal investment in mammals. (Translated from eng) *Am. Nat.* 177(1):86-98 (in eng).

24. Reep RL, Finlay BL, & Darlington RB (2007) The limbic system in Mammalian brain evolution. (Translated from eng) *Brain Behav. Evol.* 70(1):57-70 (in eng).

25. Smuts BB, Cheney DL, Seyfarth RM, & Wrangham RW (1987) *Primate Societies* (University of Chicago Press).

26. Crile G & Quiring DP (1940) *A Record of the Body Weight and Certain Organ and Gland Weights of 3690 Animals* (Ohio Journal of Science).

27. Lahann P (2007) Biology of Cheirogaleus major in a littoral rain forest in southeast madagascar. (Translated from English) *Int J Primatol* 28(4):895-905 (in English).

28. Barrickman NL & Lin MJ (2010) Encephalization, expensive tissues, and energetics: An examination of the relative costs of brain size in strepsirrhines. (Translated from eng) *Am. J. Phys. Anthropol.* 143(4):579-590 (in eng).

29. Sherwood CC*, et al.* (2009) Neocortical neuron types in Xenarthra and Afrotheria: implications for brain evolution in mammals. (Translated from eng) *Brain Struct. Funct.* 213(3):301-328 (in eng).

30. Manger PR, Hemingway J, Haagensen M, & Gilissen E (2010) Cross-sectional area of the elephant corpus callosum: comparison to other eutherian mammals. (Translated from eng) *Neuroscience* 167(3):815-824 (in eng).

31. Knoll A (2012) Crocidura russula (On-line).).

32. Taylor MJ & Hoffmann M (1993) Cynictis penicillata. *Mammalian Species* (432):1-7.

33. Wenhold OAE, Howard BA, Marais P, Pallet A, & Rasa J (1992) Reproduction in the Yellow Mongoose Revisited. *South African Journal of Zoology* 27(4):192-195.

34. Bricklin R (2004.) Dasyprocta leporina (On-line). in *Animal Diversity Web*).

35. Food & Mammals AOotUNWPoM (1981) *Mammals in the Seas: General papers and large Cetaceans* (FAO).

36. Alspaugh M (2000) Delphinus delphis (On-line). in *Animal Diversity Web*).

37. Marino L, Murphy TL, Gozal L, & Johnson JI (2001) Magnetic resonance imaging and three-dimensional reconstructions of the brain of a fetal common dolphin, Delphinus delphis. (Translated from eng) *Anat. Embryol.* 203(5):393-402 (in eng).

38. Weisbecker V & Goswami A (2010) Brain size, life history, and metabolism at the marsupial/placental dichotomy. (Translated from eng) *Proc. Natl. Acad. Sci. U. S. A.* 107(37):16216-16221 (in eng).

39. Roberts C (2011) Erinaceus europaeus. in *Animal Diversity Web*).

40. Curtis DJ (2004) Diet and nutrition in wild mongoose lemurs (Eulemur mongoz) and their implications for the evolution of female dominance and small group size in lemurs. (Translated from eng) *Am. J. Phys. Anthropol.* 124(3):234-247 (in eng).

41. Nowak RM, Mittermeier RA, Rylands AB, & Konstant WR (1999) *Walker's Primates of the World* (Johns Hopkins University Press).

42. Williams RW, Cavada C, & Reinoso-Suarez F (1993) Rapid evolution of the visual system: a cellular assay of the retina and dorsal lateral geniculate nucleus of the Spanish wildcat and the domestic cat. (Translated from eng) *J. Neurosci.* 13(1):208-228 (in eng).

43. Clancy B, Darlington RB, & Finlay BL (2001) Translating developmental time across mammalian species. *Neuroscience* 105(1):7-17.

44. Roberts M (1994) Growth, Development, and Parental Care in the Western Tarsier (Tarsius-Bancanus) in Captivity - Evidence for a Slow Life-History and Nonmonogamous Mating System. (Translated from English) *Int J Primatol* 15(1):1-28 (in English).

45. Ballenger L (2001) Galago senegalensis (On-line). in *Animal Diversity Web*).

46. Manger PR (2006) An examination of cetacean brain structure with a novel hypothesis correlating thermogenesis to the evolution of a big brain. (Translated from English) *Biological Reviews* 81(2):293-338 (in English).

47. Mutschler T, Feistner AT, & Nievergelt CM (1998) Preliminary Field Data on Group Size, Diet and Activity in the Alaotran Gentle Lemur Hapalemur griseus alaotrensis. (Translated from Eng) *Folia Primatol (Basel)* 69(5):325-330 (in Eng).

48. Mutschler T, Nievergelt CM, & Feistner AT (2000) Social organization of the Alaotran gentle lemur (Hapalemur griseus alaotrensis). (Translated from eng) *Am J Primatol* 50(1):9-24 (in eng).

49. Frens K (2009) Hydrochaerus hydrochaeris (On-line). in *Animal Diversity Web*).

50. Rowe N (1996) *The pictorial guide to the living primates* (Pogonias Press).

51. Axel AC & Maurer BA (2011) Lemurs in a Complex Landscape: Mapping Species Density in Subtropical Dry Forests of Southwestern Madagascar Using Data at Multiple Levels. (Translated from English) *Am J Primatol* 73(1):38-52 (in English).

52. Rasoloharijaona S, Randrianambinina B, & Zimmermann E (2008) Sleeping site ecology in a rain-forest dwelling nocturnal lemur (Lepilemur mustelinus): implications for sociality and conservation. (Translated from eng) *Am J Primatol* 70(3):247-253 (in eng).

53. Hilgartner R, Zinner D, & Kappeler PM (2008) Life history traits and parental care in Lepilemur ruficaudatus. (Translated from English) *Am J Primatol* 70(1):2-11 (in English).

54. Shoshani J, Kupsky WJ, & Marchant GH (2006) Elephant brain. Part I: gross morphology, functions, comparative anatomy, and evolution. (Translated from eng) *Brain Res Bull* 70(2):124-157 (in eng).

55. Hakeem AY*, et al.* (2005) Brain of the African elephant (Loxodonta africana): neuroanatomy from magnetic resonance images. (Translated from eng) *Anat. Rec. A Discov. Mol. Cell. Evol. Biol.* 287(1):1117-1127 (in eng).

56. Clancy B, Teague-Ross TJ, & Nagarajan R (2009) Cross-species analyses of the cortical GABAergic and subplate neural populations. (Translated from eng) *Front. Neuroanat.* 3:20 (in eng).

57. Miller D (2002) Macropus fuliginosus (On-line). in *Animal Diversity Web*).

58. Byers JA (1999) The distribution of play behaviour among Australian marsupials. (Translated from English) *J Zool* 247:349-356 (in English).

59. Champagne A (2006) Mesocricetus auratus (On-line). in *Animal Diversity Web*).

60. Seelke AM, Dooley JC, & Krubitzer LA (2013) Differential changes in the cellular composition of the developing marsupial brain. (Translated from eng) *J. Comp. Neurol.* 521(11):2602-2620 (in eng).

61. Seelke AM, Dooley JC, & Krubitzer LA (2014) The cellular composition of the marsupial neocortex. (Translated from eng) *J. Comp. Neurol.* 522(10):2286-2298 (in eng).

62. Ballenger L (1999) Mus musculus (On-line). in *Animal Diversity Web*).

63. Haubensak W, Attardo A, Denk W, & Huttner WB (2004) Neurons arise in the basal neuroepithelium of the early mammalian telencephalon: A major site of neurogenesis. *Proc. Natl. Acad. Sci. USA* 101:3196-3201.

64. Kowalczyk T*, et al.* (2009) Intermediate neuronal progenitors (basal progenitors) produce pyramidal-projection neurons for all layers of cerebral cortex. (Translated from Eng) *Cereb. Cortex* 19(10):2439-2450 (in Eng).

65. Jones EG & Diamond IT (1995) *Cerebral Cortex: Volume 11: The Barrel Cortex of Rodents* (Springer).

66. Tislerics A (2000) Oryctolagus cuniculus (On-line). in *Animal Diversity Web*).

67. Ebinger P (1975) Quantitative investigations of visual brain structures in wild and domestic sheep. (Translated from eng) *Anatomy and embryology* 146(3):313-323 (in eng).

68. Bininda-Emonds ORP (2000) Pinniped brain sizes. (Translated from English) *Marine Mammal Science* 16(2):469-481 (in English).

69. Marino L (1999) Brain growth in the harbor porpoise and Pacific white-sided dolphin. (Translated from English) *J Mammal* 80(4):1353-1360 (in English).

70. Olds N & Shoshani J (1982) Procavia capensis. *Mammalian Species* (171):1-7.

71. Linderman E (2011) Procavia capensis (On-line). in *Animal Diversity Web*).

72. Dunbar RIM & Bever J (1998) Neocortex size predicts group size in carnivores and some insectivores. (Translated from English) *Ethology* 104(8):695-708 (in English).

73. Manger PR, Prowse M, Haagensen M, & Hemingway J (2012) Quantitative analysis of neocortical gyrencephaly in African elephants (Loxodonta africana) and six species of cetaceans: comparison with other mammals. (Translated from eng) *J. Comp. Neurol.* 520(11):2430-2439 (in eng).

74. Silbernagel E (2005) Pteropus giganteus (On-line). in *Animal Diversity Web*).

75. Jurgens KD & Prothero J (1987) Scaling of maximal lifespan in bats. (Translated from eng) *Comp Biochem Physiol A Comp Physiol* 88(2):361-317 (in eng).

76. Marshall AJ (1947) The breeding cycle of an equatorial bat (Pteropus giganteus of Ceylon). *Proceedings of the Linnean Society of London* 159(2):103-111.

77. Hosken DJ (1998) Testes mass in megachiropteran bats varies in accordance with sperm competition theory. (Translated from English) *Behav Ecol Sociobiol* 44(3):169-177 (in English).

78. Armitage D (2004) Rattus norvegicus (On-line). in *Animal Diversity Web*).

79. Kruska D & Rohrs M (1974) Comparative--quantitative investigations on brains of feral pigs from the Galapagos Islands and of European domestic pigs. (Translated from eng) *Z. Anat. Entwicklungsgesch.* 144(1):61-73 (in eng).

80. Sondergaard E (2006) Talpa europaea (On-line). in *Animal Diversity Web*).

81. Smith RJ & Leigh SR (1998) Sexual dimorphism in primate neonatal body mass. *J. Hum. Evol.* 34(2):173-201.

82. Dagosto M, Gebo D, & Dolino C (2001) Positional behavior and social organization of the philippine tarsier (&lt;i&gt;Tarsius syrichta&lt;/i&gt;). *Primates* 42(3):233-243.

83. Reep RL & O'Shea TJ (1990) Regional brain morphometry and lissencephaly in the Sirenia. (Translated from eng) *Brain Behav. Evol.* 35(4):185-194 (in eng).

84. Edwards H (2000) Trichechus manatus (On-line). in *Animal Diversity Web*).

85. Herculano-Houzel S, Collins CE, Wong P, & Kaas JH (2007) Cellular scaling rules for primate brains. (Translated from eng) *Proc. Natl. Acad. Sci. U. S. A.* 104(9):3562-3567 (in eng).

86. Kawamichi T & Kawamichi M (1979) Spatial-Organization and Territory of Tree Shrews (Tupaia-Glis). (Translated from English) *Animal Behaviour* 27(May):381-393 (in English).

87. Cisneros L (2005) Tupaia glis (On-line). in *Animal Diversity Web*).

88. Jenkins J (2009) Tursiops truncatus (On-line). in *Animal Diversity Web*).
